# MICU1 controls spatial membrane potential gradients and guides Ca^2+^ fluxes within mitochondrial substructures

**DOI:** 10.1038/s42003-022-03606-3

**Published:** 2022-07-01

**Authors:** Benjamin Gottschalk, Zhanat Koshenov, Markus Waldeck-Weiermair, Snježana Radulović, Furkan E. Oflaz, Martin Hirtl, Olaf A. Bachkoenig, Gerd Leitinger, Roland Malli, Wolfgang F. Graier

**Affiliations:** 1 Gottfried Schatz Research Center: Molecular Biology and Biochemistry, Medical University of Graz, Graz, Austria; 2 Gottfried Schatz Research Center: Cell Biology, Histology and Embryology, Medical University of Graz, Graz, Austria; 3 BioTechMed Graz, Graz, Austria

**Keywords:** Mitochondria, Calcium signalling

## Abstract

Mitochondrial ultrastructure represents a pinnacle of form and function, with the inner mitochondrial membrane (IMM) forming isolated pockets of cristae membrane (CM), separated from the inner-boundary membrane (IBM) by cristae junctions (CJ). Applying structured illumination and electron microscopy, a novel and fundamental function of MICU1 in mediating Ca^2+^ control over spatial membrane potential gradients (SMPGs) between CM and IMS was identified. We unveiled alterations of SMPGs by transient CJ openings when Ca^2+^ binds to MICU1 resulting in spatial cristae depolarization. This Ca^2+^/MICU1-mediated plasticity of the CJ further provides the mechanistic bedrock of the biphasic mitochondrial Ca^2+^ uptake kinetics via the mitochondrial Ca^2+^ uniporter (MCU) during intracellular Ca^2+^ release: *Initially*, high Ca^2+^ opens CJ via Ca^2+^/MICU1 and allows instant Ca^2+^ uptake across the CM through constantly active MCU. *Second*, MCU disseminates into the IBM, thus establishing Ca^2+^ uptake across the IBM that circumvents the CM. Under the condition of MICU1 methylation by PRMT1 in aging or cancer, UCP2 that binds to methylated MICU1 destabilizes CJ, disrupts SMPGs, and facilitates fast Ca^2+^ uptake via the CM.

## Abstract (web summary)

Within mitochondrial substructures, MICU1 is shown to mediate Ca^2+^ control over spatial membrane potential gradients (SMPGs), with alterations in SMPGs following Ca^2+^ binding to MICU1 resulting in spatial cristae depolarization.

## Introduction

The ultrastructure of the inner mitochondrial membrane (IMM) is separated into two parts, the inner boundary membrane (IBM) representing the interface of the IMM with the outer mitochondrial membrane (OMM), and the cristae membrane (CM), forming the membrane invaginations into the mitochondrial matrix^1^. Morphologically, the cristae junctions (CJ), bottleneck membrane structures with a diameter of approximately 17 nm^2,3^, separate IBM and CM from each other. The mitochondrial contact site and cristae organizing system (MICOS) proteins and the scaffold protein optic atrophy 1 (OPA1) stabilize the CJ^4^.

All three IMM compartments CJ, IBM, and CM differ in morphology and composition of characteristic proteins according to their spatio-specific functions. In particular, the IBM hosts the mitochondrial protein uptake complex TIM (translocase of the inner membrane) that interacts with the TOM (translocase of the outer membrane) complex in the OMM to translocate nuclear-encoded mitochondrial proteins into the mitochondrial matrix or mitochondrial membranes^5^. The CM contains the four complexes of the respiratory chain^6^. Of those, complex I, III, and IV exhibit proton pump activity and, thus, contribute to the mitochondrial membrane potential (ΔΨ_m_) across the IMM. The ΔΨ_m_ represents the energetic force for the production of ATP by the F_0_F_1_ ATPase that is also localized to the CM^7^. By knowing the spatial restriction of complexes I, III and IV to the CM, experimentally determined differences in pH of CM (pH 7.0)^8^ and mathematically estimated pH values at the IBM (pH 7.4)^9^ become comprehensible. The CJ, representing a physical diffusion barrier, constrains the diffusion of the protons to the cristae lumen. Rieger et al. showed that a pH gradient is even established between the Complex IV and the F_0_F_1_ ATPase in the isolated CM compartment^8^. The proton motive force towards the matrix is the main component contributing to establish the ΔΨ_m_^10^. Wolf et al. showed recently that individual cristae are isolated insulators restricting the membrane potential generated at the CM by the CJ. This study demonstrated two distinct membrane potentials at the CM (ΔΨ_CM_) and the IBM (ΔΨ_IBM_)^11^.

Mitochondrial Ca^2+^ uptake, which highly depends on ΔΨ, is essential to activate Ca^2+^-dependent dehydrogenases of the TCA cycle and increase oxidative phosphorylation^12^. Mitochondrial Ca^2+^ Uniporter (MCU) complex-mediated Ca^2+^ uptake is driven by the ΔΨ_m_ across the IMM, yet it was never elaborated if the different CM (ΔΨ_CM_) and the IBM (ΔΨ_IBM_) potentials influence the Ca^2+^ uptake on the level of mitochondrial ultrastructure.

The main regulator of MCU, mitochondrial Ca^2+^ uptake 1 (MICU1), is localized at the IBM, stabilizes the CJ, and interacts with the MICOS complex^2,13^. Under basal conditions, MICU1 exists as a hexamer or oligomer, which in case of Ca^2+^ binding via EF-hands of MICU1 disassembles into dimers with an EC_50_ of 3.8 μM Ca^2+^^14,15^. Methylation of MICU1 by protein arginine methyltransferase 1 (PRMT1) was shown to reduce the Ca^2+^-binding affinity of MICU1 to approximately 18.5 µM and inhibit thereby the Ca^2+^ induced dissociation into dimers. Contrary, the specific binding of uncoupling protein 2 (UCP2) to methylated MICU1 counteracts the methylation effect and resensitizes MICU1 towards Ca^2+^ (EC_50_ = 4.0)^15^. Intracellular ER Ca^2+^ release from the endoplasmic reticulum (ER) leads to OMM located Ca^2+^ hotspots (up to 16.42 µM) in close proximity to mitochondrial-associated ER membranes (MAM), while mitochondrial regions without direct contact with the ER are facing much lower Ca^2+^ concentration of approximately 2.94 µM^16^. The precise regulation of MICU1 by PRMT1 and UCP2 is important for cell metabolism and viability^17^. Especially cancer cells show a clear increase in UCP2 but also PRMT1 expression and both proteins when highly expressed are prognostic markers for lung carcinoma patients^18^.

The ability of MICU1 to respond to Ca^2+^ hotspots with changes in its quaternary structure paired with the observation that MICU1 stabilizes CJ led us to hypothesize that the opening and stability of the CJ are directly regulated by high Ca^2+^ ^19^. Strong opening of the CJ is associated with a loss of membrane potential^2,11^, impaired oxidative phosphorylation and ATP production^20,21^, and induction of apoptosis^22^. Accordingly, we used structured illumination microscopy, differential membrane potential measurements of the CM and IBM, electron microscopy, and mitochondria-targeted genetically encoded biosensors for Ca^2+^ to elaborate the involvement of MICU1 in CJ dynamics, effects on mitochondrial Ca^2+^ uptake, and ΔΨ_m_ homeostasis between IBM and CM.

## Results

### Spatial membrane potential distributions exist between CM and IBM

To detect spatial membrane potential gradients (SMPGs) between the CM (ΔΨ_CM_) and the IBM (ΔΨ_IBM_), we established a reliable and robust method using super-resolution microscopy. This approach builds on the assumption that the fluorescent potentiometric dye tetramethylrhodamine methyl ester (TMRM) is incorporated into the IMM^23–25^ according to local ΔΨ, thus, the individual fluorescent intensities reflect distinct ΔΨ_IBM_ and ΔΨ_CM_. While at low and moderate concentrations TMRM binds to the IMM in a linear correlation with the ΔΨ_m_, saturation effects have been reported at high TMRM concentrations^21^. Further, we could show that TMRM concentrations in the range of 1.35–81 nM show a slight saturation effect (Supplementary Fig. 1). Therefore, we assumed that at a given high concentration TMRM gets saturated in the CM while the TMRM accumulation maintains in a linear correlation in the IBM with its lower ΔΨ. Thus, we hypothesized that both sub-mitochondrial compartments vary in their labeling properties and the shifted TMRM affinity can be used to distinguish the different membrane potentials of the two sub-compartments of the IMM.

At very high concentrations of TMRM, self-quenching effects are known^25^. Therefore, we measured concentrations of 1.35, 13.5, 81, 200, 500, and 1000 nM TMRM for self-quenching effects. For 1.35–81 nM no self-quenching effects were observed and only at 500–1000 nM TMRM we measured considerable self-quenching (Supplementary Fig. 2), as reported elsewhere^26,27^. Accordingly, the used range of 1.35–81 nM TMRM used to detect mitochondrial membrane potential gradients was not affected by TMRM self-quenching effects.

To ensure that TMRM is specifically labeling and accumulating in the IMM, HeLa cells expressing mitochondrial matrix targeted superfolder green fluorescent protein (mt-sfGFP) and stained with 81 nM TMRM were treated with 100 µM ATP to induce mitochondrial swelling. A clear separation of TMRM cristae labeling and matrix mt-sfGFP fluorescence became obvious, showing specific labeling of the IMM by TMRM (Supplementary Fig. 3), as shown by others^23,24^.

**Fig. 1:**
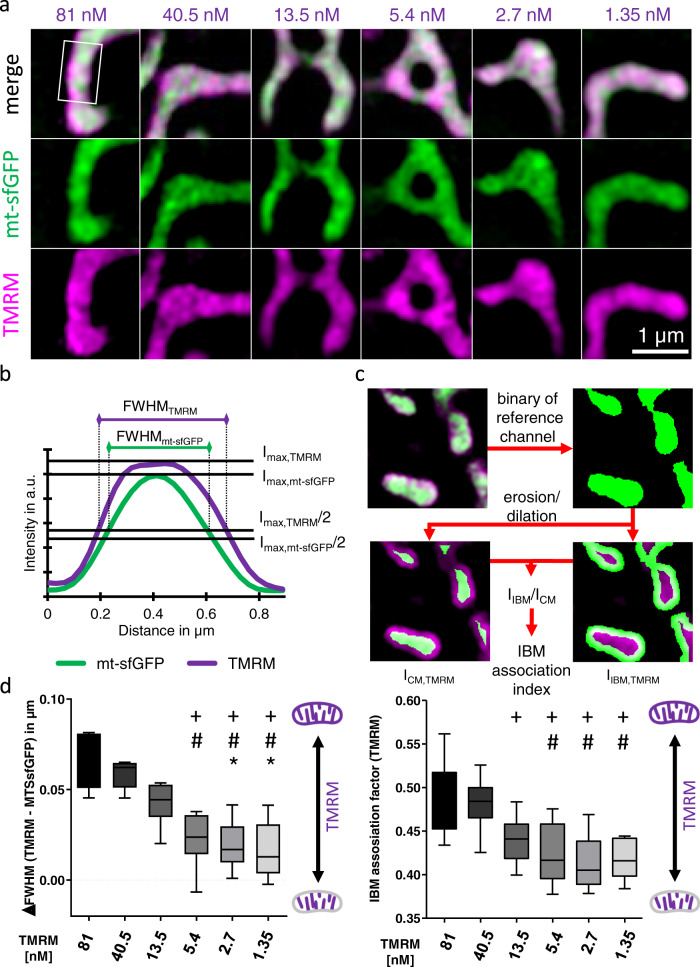
Measuring cristae and inner boundary membrane potential gradient. **a** Representative images of HeLa cells expressing mt-sfGFP (green), stained with 81, 40.5, 13.5, 5.4, 2.7, or 1.35 nM tetramethylrhodamine methyl ester (TMRM) (magenta) and examined using simultaneous dual-color 3D-SIM. **b** Intensity line plots of the mitochondrion in the white box in **a** showing the mt-sfGFP and TMRM intensity distribution. Full width at half maximum (FWHM) of mt-sfGFP and TMRM distributions are indicated with the corresponding maximal (*I*_max_) and half-maximal (*I*_max_/2) intensities. **c** Schematic illustration of the calculation of the IBM association index on the example of HeLa cells expressing mt-sfGFP (green) and stained with 81 nM TMRM (magenta). The reference channel (mt-sfGFP) is thresholded and split by erosion and dilation into inner boundary membrane (IBM) and cristae membrane (CM) related segments, which are used as masks to measure TMRM mean intensities. The ratio of IBM intensity (*I*_IBM,TMRM_) and CM intensity (*I*_CM,TMRM_) results in the IBM association index. **d** Quantitative analysis of **a** using the ∆FWHM of TMRM and mt-sfGFP (described in **b**) or the IBM association index (described in **c**) to determine TMRM distribution to the IBM. The higher ∆FWHM or IBM association index, the broader the TMRM distribution indicating a stronger TMRM staining in the IBM. Data information: Horizontal lines in **d** represent the median, the lower and upper hinge show, respectively, first quartile and third quartile, and lower and upper whiskers encompass minimal and maximal values. Images and analyses were obtained from each 9–10 cells in 6 independent experimental days (*n* = 6). *P < 0.05 vs. 13.5 nM, ^#^*P* < 0.05 vs. 40.5 nM and ^+^*P* < 0.05 vs. 81 nM TMRM conditions evaluated using one-way analysis of variance (ANOVA) with Bonferroni post hoc test.

In a first attempt, we investigated the sub-mitochondrial localization of TMRM and mt-sfGFP using super-resolution dual-color structured illumination microscopy (SIM). At low TMRM concentrations (≤5.4 nM) the overlay of TMRM with mt-sfGFP shows an exclusive TMRM staining of the cristae membrane (Fig. 1a). At TMRM concentrations ≥13.5 nM an additional halo of TMRM fluorescence surrounding the matrix develops (Fig. 1a), pointing to stronger labeling of the inner boundary membrane (IBM).

Two different methods were used to quantify the spatial distribution of TMRM in relation to the reference channel mt-sfGFP: (1) The full width of half maxima (FWHM) of mitochondria for the TMRM and mt-sfGFP (i.e., matrix) channels were measured and their differences (ΔFWHM_(TMRM − __mt-sfGFP)_) illustrate the concentration-dependent TMRM expansion (Fig. 1b). (2) The IBM association index (AI_IBM_)^2^, which correlates the TMRM fluorescence intensities of the central cristae membrane (CM) with the IBM (Fig. 1c). For both parameters, ΔFWHM_(TMRM − mt-sfGFP)_ and AI_IBM_, a sigmoidal correlation between a TMRM accumulation in the mitochondrial periphery (halo) and increasing TMRM concentration was found, indicating different membrane potentials at the CM and IBM (Fig. 1d).

### MICU1 and OPA1 contribute to the isolation of CM and IBM potentials

**Fig. 2:**
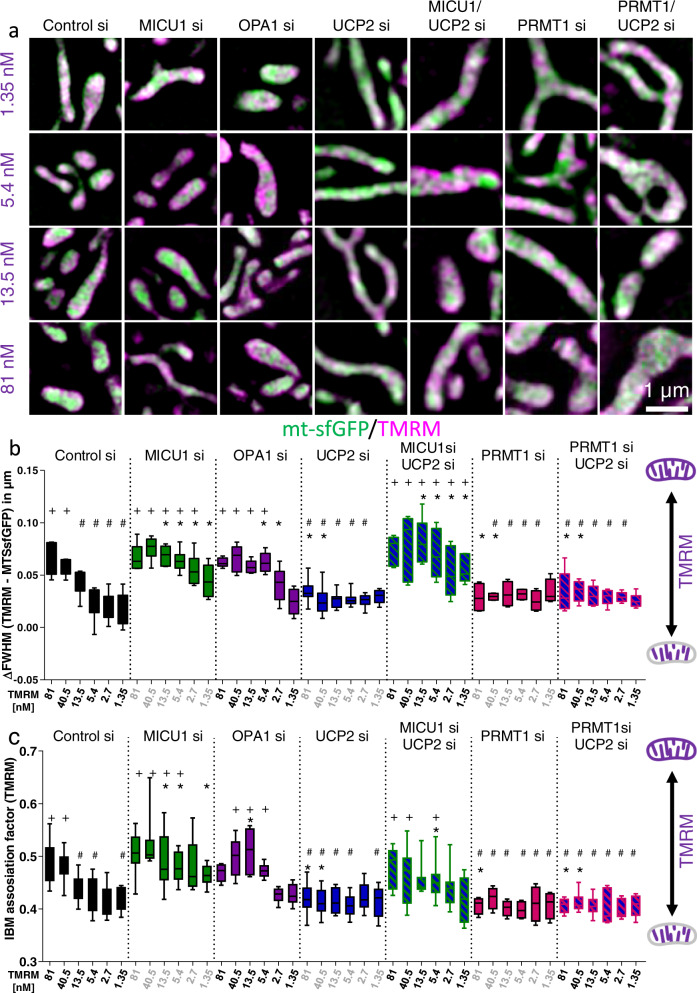
Cristae and inner boundary membrane potential gradient are controlled by MICU1, UCP2, and OPA1. **a** Representative images of HeLa cells expressing mt-sfGFP (green) and single or double transfected with Control, MICU1, OPA1, UCP2, or PRMT1 siRNA, stained with 81, 13.5, 5.4, or 1.35 nM tetramethylrhodamine methyl ester (TMRM) (magenta) and examined using simultaneous dual-color 3D-SIM. **b** Quantitative analysis of TMRM concentrations presented in **a** plus 40.5 and 2.7 nM TMRM concentration using the ∆FWHM of TMRM and mt-sfGFP to determine TMRM association with the IBM. The higher ∆FWHM, the broader the TMRM distribution indicating a stronger TMRM staining in the IBM. **c** Quantitative analysis of the same TMRM concentrations used in **b** using the IBM association factor of TMRM to determine TMRM association with the IBM. The relation of the IBM association factor to the TMRM distribution is similar to that of ∆FWHM **b**. Data information: horizontal lines represent the median, the lower and upper hinge show respectively first quartile and third quartile, and lower and upper whiskers encompass minimal and maximal values. Images and analyses were obtained from each 9–10 cells in 5–10 independent experimental days (*n*_Control si_ = 8, *n*_MICU1 si_ = 7, *n*_OPA1 si_ =  5, *n*_UCP2 si_ = 10, *n*_MICU1 si /UCP2 si_ = 7, *n*_PRMT1 si_ = 6, *n*_PRMT1 si / UCP2 si_ = 6). *P < 0.05 vs. respective control si ^#^*P* < 0.05 vs. respective MICU1 si and ^+^*P* < 0.05 vs. respective UCP2 si conditions carried out with two-way analysis of variance (ANOVA) and Bonferroni post hoc test.

The CJ represents the bottleneck structure separating CM and IBM, thereby enabling high proton concentration gradients across the CM^11^. MICU1^2^ and OPA1^4,28^ are known to contribute directly to the stability of the CJ. Accordingly, the contribution of these proteins to the isolation of the different mitochondrial membrane potentials (ΔΨ_m_) in CM (ΔΨ_CM_) and IBM (ΔΨ_IBM_) was investigated. Knockdown of either MICU1^29^ (Supplementary Fig. 4) or OPA1^30^ leads to an increase in IBM vs. CM staining at low TMRM concentrations (i.e. 13.5, 5.4 nM) (Fig. 2a–c and Supplementary Fig. 5), thus, indicating that an intact CJ essentially needs either MICU1 or OPA1 and is fundamental to establish a high ΔΨ_m_ restricted to the cristae. Investigations of the mitochondrial morphology revealed smaller and rounded mitochondria with lower interconnectivity in MICU1 and OPA1 depleted cells (Supplementary Fig. 6). No mitochondrial swelling was observed upon depletion of either MICU1 or OPA1 and TMRM did not affect the mitochondrial morphology (Supplementary Fig. 7).

Because UCP2 was recently shown to interact with methylated MICU1 and, thereby, pseudo-normalizing the Ca^2+^ sensitivity and Ca^2+^ dependent quaternary structure of methylated MICU1^15^, we next tested the roles of UCP2 and PRMT1 in CJ stability. As indicated by the low ΔFWHM_(TMRM − mt-sfGFP)_ and AI_IBM_, knockdown of UCP2^18,31^ results in the accumulation of TMRM in the CM for all TMRM concentrations applied (Fig. 2a–c and Supplementary Figs. 5 and 6). These data indicate that the lack of UCP2 yields a hyperpolarization of the CM vs. IBM. Based on our previous observations^2,15^ it is tempting to speculate that UCP2 knockdown strengthens CM isolation from the IBM, since UCP2 does not interfere with methylated MICU1. This assumption is further supported by our findings that double knockdown of UCP2 and MICU1 prevented CM hyperpolarization upon UCP2 depletion (Fig. 2a–c and Supplementary Figs. 5 and 6), thus, pointing to a MICU1-dependent role of UCP2.

Next, we tested the influence of PRMT1 silencing^18^ on the ΔΨ_m_ distribution. PRMT1 knockdown lead to a clear clustering of ΔΨ at the CM (Fig. 2a–c and Supplementary Figs. 5 and 6), confirming the exclusive function of UCP2 on methylated MICU1. Accordingly, experimental results from cells that were depleted from UCP2 and PRMT1 were not different from a single knockdown experiment (Fig. 2a–c and Supplementary Figs. 5 and 6). Structurally, knockdown of UCP2 and PRMT1 lead to higher mitochondrial interconnectivity and elongated shape while mitochondrial thickness did not substantially change (Supplementary Fig. 7).

### MICU1, UCP2, and OPA1 knockdown influences spatial CM distribution

**Fig. 3:**
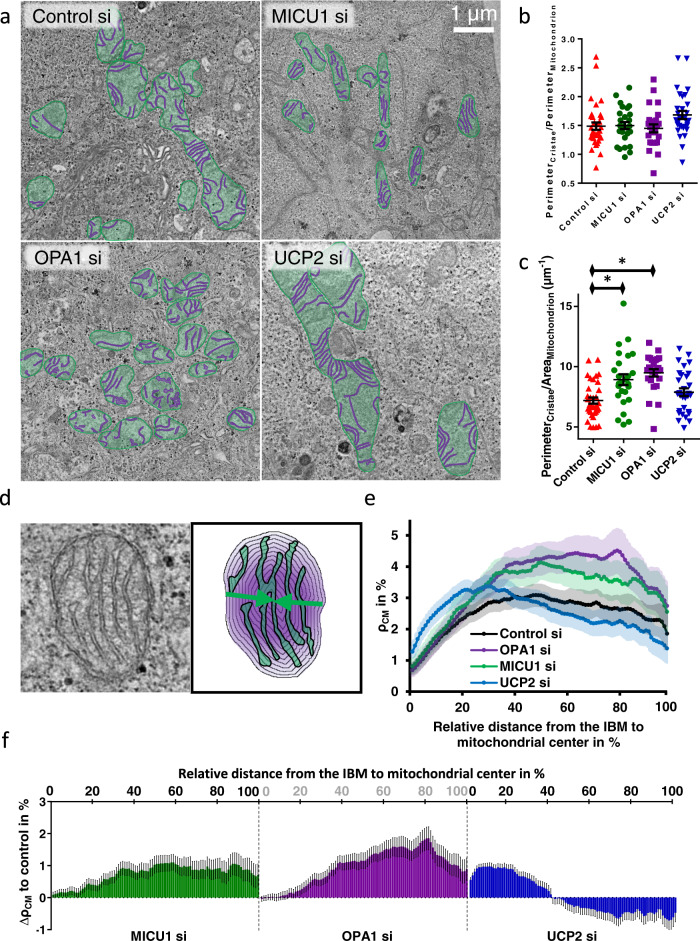
Knockdown of MICU1, UCP2, and OPA1 are influencing mitochondrial cristae membrane distribution. **a** TEM images of mitochondria of HeLa cells transfected with Control, MICU1, OPA1, or UCP2 siRNA. Mitochondria are highlighted in green and cristae are highlighted in magenta. **b** The amount of CM was quantified as the perimeter of cristae and normalized to the respective mitochondrial perimeter per cell. **c** Cristae density was calculated by normalizing the cristae perimeter to the mitochondrial area. **d** Schematic illustration of the measurement of spatial cristae density within mitochondria. After segmentation of mitochondria and the cristae, iterative measurements of the cristae membrane density in gradually downsized circular segments were conducted. **e** TEM images of mitochondria of HeLa cells transfected with Control siRNA (Control si) or siRNA against MICU1 (MICU1 si) or OPA1 (OPA1 si) were analyzed in regard to the spatial cristae density (ρ_CM_) with the methods as displayed in **d**. On the y-axis 0 represents the most outer shell of the mitochondrion while 100 represents the mitochondrial center. Data are shown as the mean +/− 95% Confidence interval. **f** For better illustration the delta cristae density (∆ρ_CM_) to control was calculated for MICU1 and OPA1 knockdown cells. ∆ρ_CM_ to control siRNA-treated cells of MICU1 and OPA1-depleted cells were plotted in relation to the relative distance from the IBM. Data information: data in **b**, **c** are shown as dot plots representing individual cells with the mean +/− SEM as middle line and whiskers, respectively (*n*_Control si_ = 35, *n*_MICU1 si_ = 26, *n*_OPA1 si_ = 25, *n*_UCP2 si_ = 30) (**a**–**c**). Data in **e**, **f** are shown as mean +/− SEM of individual mitochondria (*n*_Control si_ = 3/35/82, *n*_MICU1 si_ = 2/26/91, *n*_OPA1 si_ = 2/26/123, *n*_UCP2 si_ = 2/30/73 with preparations/cells/mitochondria). For **f**, the SEM was determined by Gaussian error propagation. *P < 0.05 vs. respective control conditions carried out with one-way analysis of variance (ANOVA) with Bonferroni post hoc test.

Recently, we reported that a knockdown of either MICU1 or OPA1 enlarge CJ width and affect cristae morphology^2^. To verify whether changes in IBM/CM TMRM ratio manifest due to the morphological changes in the IMM structure, the distribution of CM across the entire mitochondrial diameter was evaluated in MICU1, OPA1 or UCP2 depleted cells with transmission electron microscopy (Fig. 3a). MICU1 or OPA1 knockdown did not affect the ratio of the OMM to CM length (Fig. 3b). However, normalizing the cristae perimeter to the mitochondrial area, unveiled increased cristae density in OPA1 knockdown cells (Fig. 3c). OPA1 and MICU1 knockdown lead to mitochondrial fragmentation (Supplementary Fig. 7) while the relative amount of CM in the mitochondria remains unchanged (Fig. 3b). The increased cristae density in OPA1 and MICU1 knockdown cells most likely relates to the decreased mitochondrial area/perimeter ratio. Silencing of UCP2 did not change cristae density.

However, the IBM/CM TMRM intensity ratios may also be influenced by the spatial distribution of CM within mitochondria. To evaluate this possibility, consecutive circular segments of mitochondria (shown in Fig. 3d) were analyzed in regard to the cristae density (ρ_CM_). Both, MICU1 and OPA1 depletion result in a focused elevation of ρ_CM_ in the mitochondrial center (Fig. 3e, f). Despite central clustering of IMM upon depletion of OPA1 or MICU1, both knockdowns lead to higher ΔFWHM_(TMRM – mt-sfGFP)_ and IBM association index (Fig. 2b, c). Because direct adaption of spatial membrane quantity would have led to increased CM fluorescence intensity under MICU1 or OPA1 knockdown, these results indicate that the spatial ρ_CM_ distribution does not disturb the super-resolution measurements of ΔΨ_IBM_/ΔΨ_CM_. The knockdown of UCP2 causes a strong increase of ρ_CM_ in the mitochondrial periphery (Fig. 3e, f), most likely derived from an increased number of CJ (Supplementary Fig. 8).

Additionally, we evaluated whether or not the addition of TMRM in the various concentrations influences the cristae morphology or density. Neither the amount of CM nor ρ_CM_ was changed while the spatial distribution of ρ_CM_ was slightly elevated in the mitochondrial center with increased TMRM concentrations (Supplementary Fig. 9).

### Ca^2+^ activation of MICU1 triggers a transient CJ opening and spatial cristae depolarization

**Fig. 4:**
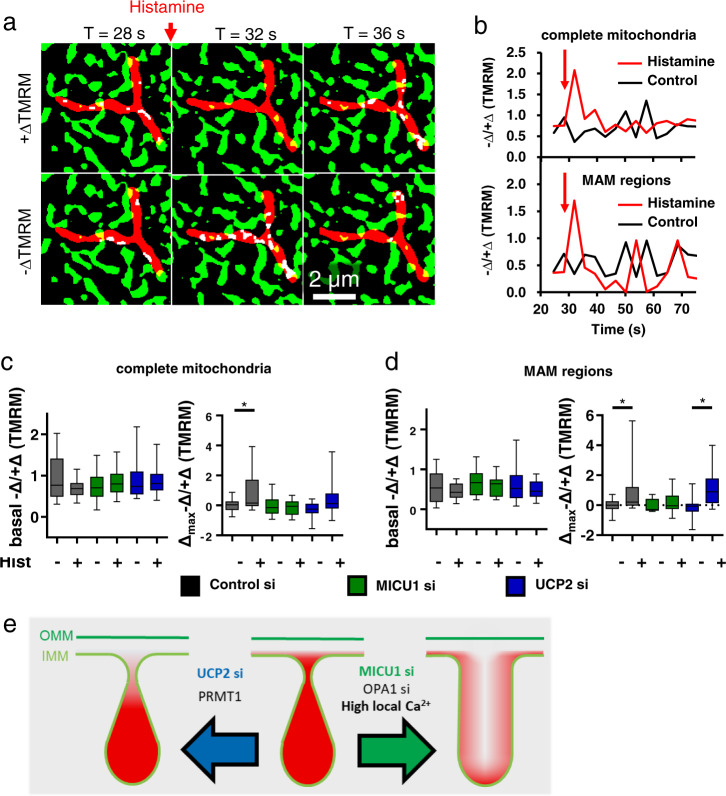
Spatial loss of ΔΨ_m_ after ER Ca^2+^ release is modulated by MICU1 and UCP2. **a** Binarized labeling of HeLa cells at *T* = 28 s, 32 s, and 36 s showing thresholded mitochondrial (TMRM; red), endoplasmic reticulum (ERAT 4.0 NA; green) and MAMs (yellow) structures as well as positive (+∆TMRM) or negative (−∆TMRM) local TMRM intensity changes (white). **b** Quantification of spatial restricted drops in membrane potential defined as −∆/+∆TMRM over time in HeLa cells challenged with or without histamine in the complete mitochondria and (upper panel) in spatial proximity to MAMs (lower panel). The red arrows point to the addition of histamine. Statistical quantification in whole mitochondria in **c** and regions with close proximity to MAMs in **d** of basal (left panel) and maximum delta (right panel) −Δ/+ΔTMRM ratios in HeLa cell transfected with siRNA against control, MICU1, or UCP2. **e** Schematic depiction of CJ opening or closing and redistribution of local membrane potential upon knockdown of UCP2, MICU1, or OPA1 as well as the influence of PRMT1 expression and high local Ca^2+^ concentrations. Data information: data are shown as the mean +/− SEM (*n*_Control si, control_ = 9/23, *n*_Control si, hist_ = 10/29, *n*_MICU1 si, control_ = 8/24, *n*_MICU1 si, hist_ = 9/26, *n*_UCP2 si, control_ = 8/20, *n*_UCP2 si, hist_ = 9/24, with days/cells). *P < 0.05 vs. respective untreated (CaB) control conditions carried out with unpaired double-sided *T* test.

MICU1 contains two EF-hands and undergoes structural rearrangements upon Ca^2+^ binding^14^. Accordingly, we investigated the role of MICU1 in the opening of CJ upon Ca^2+^ mobilization. Therefore, HeLa cells expressing an ER marker were stained with TMRM (5.4 nM), and the respective fluorescence intensities and distributions were recorded upon stimulation with histamine (Fig. 4a), a potent IP_3_-generating agonist. In this experimental setting, we were able to measure local variations of ΔΨ_m_ depending on the proximity to the ER.

These experiments revealed that upon histamine stimulation HeLa cells react with local restricted losses of ΔΨ_m_ in IMM regions with close proximity to MAMs and punctually along the entire IMM (Fig. 4b). The local TMRM spikes with a duration of several seconds indicate restricted fluctuations of the membrane potential of single cristae in close temporal correlation to cytosolic Ca^2+^ elevations that trigger structural rearrangement of MICU1, destabilize the CJ and lead to instant local equilibration of ΔΨ_CM_ and ΔΨ_IBM_.

We have recently shown that silencing of MICU1 destabilizes the CJ^2^ and found herein that MICU1 knockdown leads to a homogenization of ΔΨ_CM_ and ΔΨ_IBM_ (Fig. 2a–c). As expected, siRNA-mediated knockdown of MICU1, and by this means absence of SMPGs, resulted in a loss of histamine-induced spatial −∆/+∆TMRM peaks (Fig. 4c).

### The role of UCP2 and Ca^2+^ hotspots on CJ integrity/stability

We previously reported that UCP2 normalizes the reduced Ca^2+^ sensitivity of PRMT1 methylated MICU1 and, thus, decreases its ability for Ca^2+^-induced fragmentation^14,15^. Knockdown of UCP2 in HeLa cells with high PRMT1 activity decreased the overall −∆/+∆TMRM response to histamine but maintained the amounts of spatial TMRM drops near MAMs (Fig. 4d). The reduced overall −∆/+∆TMRM ratio implies that the absence of UCP2 leads to a desensitization of MICU1 to Ca^2+^. Consequently, we conclude that in UCP2-depleted HeLa cells the CJ does not undergo conformational changes upon histamine stimulation, while the Ca^2+^ hotspots in the MAM region are high enough to force MICU1 deoligomerization leading to an opening of the CJ and thereby homogenizes IBM and CM potential.

Control experiments adding Ca^2+^ buffer (CaB) instead of histamine showed no response in −∆/+∆TMRM and basal −∆/+∆TMRM ratio did not change by silencing either MICU1 or UCP2 compared to control cells (Fig. 4c, d).

Store-operated Ca^2+^ entry (SOCE) after ER Ca^2+^ depletion, which leads to a homogeneous cytosolic Ca^2+^ elevation, was used to analyze −∆/+∆TMRM alterations (Supplementary Fig. 10). After Ca^2+^ re-addition no noticeable signals in −∆/+∆TMRM were detected for Control, MICU1, or UCP2 siRNA treated cells, supporting our assumption that only high local Ca^2+^ concentration can lead to the opening of the CJ and spatial depolarization of the CM **(**Fig. 4d).

### Spatial intensity of cytosolic Ca^2+^ determines sub-compartmental Ca^2+^ propagation in mitochondria

**Fig. 5:**
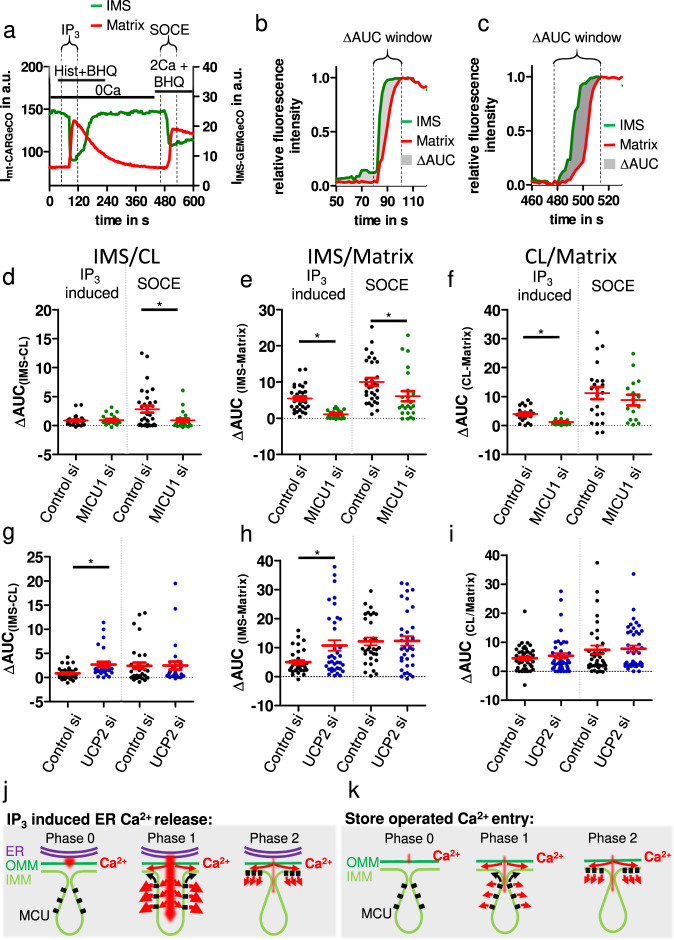
MICU1 and UCP2 modulate sub-mitochondrial Ca^2+^ transition velocity. **a** Single fluorescence intensities over time of IMS-GEMGeCO1 (green) and mt-CARGeCO1 (red) in HeLa cells upon stimulation with 100 µM histamine and 15 µM BHQ in a nominal Ca^2+^-free buffer and subsequent readdition of 2 mM extracellular Ca^2+^. **b** Normalized IMS-GEMGeCO1 (green) and mt-CARGeCO1 (red) IP_3_ induced Ca^2+^ signals with indicated ∆AUC area (gray) with upper and lower time limiting the AUC interval. **c** Normalized IMS-GEMGeCO1 (green) and mt-CARGeCO1 (red) SOCE induced Ca^2+^ signals with indicated ∆AUC area (gray) with AUC interval. ∆AUC was measured in HeLa cells treated with control or MICU1 siRNA between **d** IMS-GEMGeCO1 and CL-CARGeCO1 (*n*_IP3, Control si_ = 32; *n*_IP3, MICU1 si_ = 21; *n*_SOCE, Control si_ = 32; *n*_SOCE, MICU1 si_ = 21), **e** IMS-GEMGeCO1 and mt-CARGeCO1 (*n*_IP3, Control si_ = 31; *n*_IP3, MICU1 si_ = 23; *n*_SOCE, Control si_ = 31; *n*_SOCE, MICU1 si_ = 23), and **f** CL-GEMGeCO1 and mt-CARGeCO1 (*n*_IP3, Control si_ = 22; *n*_IP3, MICU1 si_ = 17; *n*_SOCE, Control si_ = 22; *n*_SOCE, MICU1 si_ = 16) for IP3 and SOCE induced mitochondrial Ca^2+^ uptake. ∆AUC was measured in HeLa cells treated with control or UCP2 siRNA between IMS-GEMGeCO1 and CL-CARGeCO1 **g** (*n*_IP3, Control si_ = 34; *n*_IP3,UCP2si_ = 28; *n*_SOCE, Control si_ = 34; *n*_SOCE, UCP2si_ = 28), IMS-GEMGeCO1 and mt-CARGeCO1 **h** (*n*_IP3, Control si_ = 33; *n*_IP3, UCP2si_ = 36; *n*_SOCE, Control si_ = 33; *n*_SOCE, UCP2si_ = 36), and CL-GEMGeCO1 and mt-CARGeCO1 **i** (*n*_IP3, Control si_ = 43; *n*_IP3, UCP2si_ =44n_SOCE, Control si_ = 43; *n*_SOCE, UCP2 si_ = 44) for IP_3_ and SOCE induced mitochondrial Ca^2+^ uptake. **j** IP_3_ induced ER-Ca^2+^ release leads to a biphasic Ca^2+^ uptake. (1) Ca^2+^ hotspot open the CJ and facilitate Ca^2+^ flux into the cristae from where the Ca^2+^ is taken up by constantly active MCU. (2) MCU shuttles MICU1 dependent into the IBM and enables Ca^2+^ flux into the matrix circumventing the CL. **k** Store operated Ca^2+^ entry is not opening the CJ due to the lack of Ca^2+^ hotspots in MAMs. Accordingly, the first phase of Ca^2+^ uptake is hampered resulting in a delayed Ca^2+^ uptake primarily through MCU shuttling into the IBM. Data information: data are shown as scatter plots with each dot representing one cell. The mean +/− SEM are indicated as red line and whiskers. *P < 0.05 vs. respective control conditions carried out with unpaired double-sided *T* test.

The local depolarization of CM at MAMs upon stimulation with an IP_3_-generating agonist points to a CJ opening and permeability for ions into the cristae lumen (CL). Therefore, we tested the sub-compartmental Ca^2+^ kinetics in mitochondria upon IP_3_-induced ER Ca^2+^ release and SOCE in MICU1, and UCP2 siRNA treated cells using red and green fluorescent genetically encoded Ca^2+^ biosensors targeted either to the IMS and mitochondrial matrix, CL and mitochondrial matrix, or IMS and CL, respectively. The temporal delay of Ca^2+^ propagation within the respective sub-compartment of mitochondria was quantified as the delta of areas under the curve (∆AUC) for normalized IP_3_- and SOCE-induced Ca^2+^ signals in the respective sub-compartment (Fig. 5a–c).

By silencing MICU1, the delay between the Ca^2+^ signal in the IMS and those in the CL and the matrix were reduced in SOCE- but not IP_3_-induced signals (Fig. 5d, e). The Ca^2+^ propagation from the CL into the matrix was not affected during SOCE (Fig. 5f). Thus, the Ca^2+^ propagation from the IMS to the cristae is under the control of MICU1 and the rate-limiting phase of the Ca^2+^ propagation during SOCE. Nevertheless, the increased CL and decreased matrix Ca^2+^ concentrations under SOCE in MICU1 knockdown cells indicate that MICU1 not only guards the CJ but also activates MCU-mediated Ca^2+^ influx into the matrix (Supplementary Fig. 11). Moreover, while the IP_3_-induced IMS to CL Ca^2+^-kinetics remained unaffected, the IMS to matrix and CL to matrix Ca^2+^ kinetics were clearly accelerated by MICU1 silencing (Fig. 5d–f and Supplementary Fig. 12).

Silencing of UCP2 leads to a delayed propagation of Ca^2+^ signals from the IMS to the CL and matrix after IP_3_-induced ER Ca^2+^ release. In contrast, the Ca^2+^ transition from the CL to the matrix was not affected (Fig. 5g–i and Supplementary Fig. 12). Furthermore, CL and matrix Ca^2+^ delta intensities were reduced under the knockdown of UCP2 (Supplementary Fig. 11), indicating a blockage of the CJ by UCP2 knockdown. For SOCE-induced mitochondrial Ca^2+^ uptake, UCP2 silencing did not affect the Ca^2+^ propagation between the various mitochondrial sub-compartments (Fig. 5g–i and Supplementary Fig. 12).

Taken together, these data indicate a biphasic Ca^2+^ uptake mechanism that is dependent on the origin and spatial intensity of the cytosolic Ca^2+^ signal and the stability of the CJ. In the first phase, IP_3_-induced ER Ca^2+^ release leads to cytosolic Ca^2+^ hotspot in the MAMs^16^. Subsequently, MICU1 deoligomerizes upon Ca^2+^ binding at these hotspots leading to spatial CL depolarization due to a Ca^2+^ induced opening of CJs (Fig. 4b) and consequently to the flooding of the CL with Ca^2+^, which, in turn, is instantly taken up into the matrix by constitutively active MCU within the CM. As Ca^2+^ hotspots^16^ and CJ openings (Fig. 4b) are short-lived, the second phase is dominated by the slower MICU1 mediated MCU shuttling into the IBM^2^ achieving the second phase of Ca^2+^ uptake into the mitochondrial matrix excluding the CL from the Ca^2+^ uptake process (Fig. 5j). Notably, Cytosolic Ca^2+^ signals originating from SOCE do not open the CJ due to a lack of Ca^2+^ hotspots. Thus, the propagation of SOCE signals into the mitochondrial matrix relays only on the MCU-shuttling due to partially deoligomerized MICU1 similar to the second phase in IP_3_-induced ER-Ca^2+^ release (Fig. 5k).

## Discussion

In this study, we combined dual-color SIM, TEM, and sophisticated live-cell imaging with mitochondrial sub-compartmental targeted genetically encoded biosensors to investigate the fundamental function of MICU1, OPA1, and UCP2 in the dynamic regulation of spatial membrane potential gradients (SMPGs) between IBM and CM. Moreover, the individual role of the two distinct IMM compartments (i.e. IBM and CM) in the orchestration of mitochondrial Ca^2+^ uptake has been elucidated. The CM and the IBM are isolated by the CJ that consists of the “mitochondrial contact site and cristae organizing system” (MICOS), the optic atrophy 1 (OPA1)^28,32^ and MICU1^2,13^.

Confirming recent results from ref. ^11^, we herein demonstrate that the membrane potential of the IMM (ΔΨ_IMM_) is separated by the CJ into two distinct potentials, that of the IBM (ΔΨ_IBM_) and the CM (ΔΨ_CM_). Notably, our data show that silencing of MICU1 leads to a strong shift of ΔΨ_IBM_/ΔΨ_CM_ towards the IBM. This disruption of SMPGs points to a fundamental role of MICU1 in CJ integrity, thus, reemphasizing our previous results showing a CJ opening and clear reduction of the overall ΔΨ_IMM_ for MICU1 knockdown conditions^2^. Moreover, our new data point to an alternative model of mitochondrial uncoupling that builds on the erosion of SMPGs by means of CJ-controlled ΔΨ_CM_. Opening of the CJ and the dissemination of the protons from the CL into the IMS yields depolarization of ΔΨ_CM_ that mimics a mitochondrial uncoupling across the IMM.

We found in our recent study that the CJ widened through silencing of OPA1 or MICU1^2^. We assume that a destabilization of the CJ leads to IMM constrictions which were shown to precede mitochondrial fission in ER close regions^19,33^. Our findings in this study suggest that mitochondrial fragmentation observed in MICU1 and OPA1 silenced cells is caused by destabilized CJ in ER close regions leading to IMM constriction and mitochondrial fission. Contrary reduction of UCP2 leads to a more stable CJ configuration^19^ and thus to elongated mitochondria.

Because silencing of MICU1 destabilizes CJ under basal conditions and yields disruption of SMPGs, we speculate that even under resting condition, basal ER-originated Ca^2+^cycling within the MAMs achieves rare activation of MICU1 and, thus, increase in H^+^-permeability of the CJ. Besides the process of Ca^2+^ cycling, CJ H^+^-permeability is further influenced by the methylation status of MICU1 by PRMT1. Methylation of MICU1 lowers the Ca^2+^ binding affinity of MICU1 (EC_50_ for Ca^2+^ increases from 3.8 to 18.5 µM)^16^ resulting in an enhanced stability/impermeability of the CJ. However, UCP2 binds exclusively to methylated MICU1, restores Ca^2+^ sensitivity of methylated MICU1 (EC_50_ = 4.0 µM)^15^, and *pseudo-*normalizes basal CJ H^+^-permeability. Consistently, in the present work, depletion of UCP2 resulted in an increased CJ stability due to the low Ca^2+^ binding affinity of methylated MICU1 that cannot be activated by basal ER-originated Ca^2+^ cycling within the MAMs in the absence of UCP2. In line with our findings, UCP2 knockdown is reported to increase ΔΨ_m_^34^, a phenomenon that, based on our present data, can be attributed to a stabilization of the CJ.

Under the condition of cell stimulation that triggers IP_3_-induced intracellular Ca^2+^ release, high Ca^2+^ at the mitochondrial surface binds to MICU1 yielding its deoligomerization, subsequent opening of the CJ, and disruption of individual SMPGs. Notably, the Ca^2+^ concentrations achieved within the Ca^2+^ hotspots in MAMs (up to 16.4 µM) during cell stimulation^16^, have the potential to disassemble methylated MICU1 (EC_50_ for Ca^2+^ 18.5 µM)^16^ even in the absence of UCP2. Studies focusing on cristae dynamics revealed that CJ opening is predominantly restricted to MAMs after ER Ca^2+^ release^30^ and that CJs are closely associated with MAMs^35^. In line with this assumption, Gerencer and Vizi showed that initial mitochondrial Ca^2+^ uptake after IP_3_-induced ER Ca^2+^ release originates from local foci into the whole mitochondria^36^.

Recently we reported that a knockdown of MCU and EMRE leads to an enhanced accumulation of Ca^2+^ inside the CL in response to IP_3_-induced Ca^2+^ release^26^. Further, the disruption of the CJ by knockdown of MICU1 or OPA1 increases basal Ca^2+^ inside the mitochondrial matrix^2^. Considering both findings, one may conclude that MCU is constantly active in the CM. On the other hand, MICU1 homo and MICU1/MICU2 heterodimers were shown to increase MCU conductance^37,38^ but do not occlude the MCU channel^38^. Further, three different quaternary structures including MICU1  are known : MICU1 hexamers or oligomers that disassemble Ca^2+^ dependent into MICU1 dimers^14,39^ and the MCU super complex involving MCU, EMRE, MICU1 and MICU2^40–42^. MICU1 was shown to potentiate MCU channel activity by direct interaction and MCU is not occluded by MICU1^38^, raising the question of how MICU1 reduces mitochondrial Ca^2+^ uptake at low cytosolic Ca^2+^ concentrations. These findings raise the question how MICU1 is regulating both, Ca^2+^ availability in the CM and amplification of the conductance of MCU? Accordingly, based on the present findings, a 2-phase model of the orchestration of MCU-mediated mitochondrial Ca^2+^ uptake that meets all reported features is proposed (Fig. 5j, k):

Phase 1: *High Ca*^*2+*^ *phase* with Ca^2+^ hotspots instantly after IP_3_-induced ER Ca^2+^ release:

Phase 1.1. Upon IP_3_-induced ER-Ca^2+^ release and the subsequent formation of Ca^2+^ hotspots within the MAMs, high Ca^2+^ locally disrupts CJ by deoligomerization of MICU1.

Phase 1.2. Subsequently, the CJ opens, and Ca^2+^ floats into the CL and reaches the mitochondrial matrix via constantly active MCU^38,42^ in the CM.

Phase 2: *Consolidation phase* for mitochondrial Ca^2+^ sequestration at the entire surface of the mitochondria, independently of the Ca^2+^ source:

Phase 2.1. MCU shuttling to the IBM and assembly with MICU1-dimers or MICU1-MICU2-heterodimers^2,39,43^. Importantly, the contact with MICU1/2 increases MCU conductance and further introduces MICU1/2-mediated modulation of MCU activity that is crucial to meet the Ca^2+^ micro-environment^38^. Further, the V-shaped dimeric structure of MCU, EMRE, MICU1, and MICU2 complexes is favorable for concave membranes, potentially increasing the distribution of MCU out of the convex cristae into the IBM^41,42^.

Phase 2.2. Secondary to the fast transfer of ER-derived Ca^2+^ to the mitochondria, this phase is important to manage the uptake of Ca^2+^ entering the cells via store-operated Ca^2+^ influx pathway (SOCE) that does not generate Ca^2+^ hotspots in MAMs. Accordingly, SOCE-originated mitochondrial Ca^2+^ uptake always occurs independently from UCP2^44^.

Such sophisticated organization of the Ca^2+^-triggered opening of the CJ and biphasic mitochondrial Ca^2+^ uptake ensures full mitochondrial functionality even in phases of high Ca^2+^ challenges or to achieve uptake of low Ca^2+^. Due to local and temporal isolated CM depolarization, the mitochondria maintain general stability during Ca^2+^ uptake while increasing the metabolic output. By the local regulation of CJ opening, not only Ca^2+^ uptake is regulated but also the overall ΔΨ_CM_, mostly distanced from MAMs, is protected, and only affected in a minor way. This mechanism ensures mitochondrial stability under physiological Ca^2+^ signaling.

Taken together, a model evolves by combining the separation of the mitochondrial membrane potential and the mitochondrial Ca^2+^ uptake regulation by MICU1 at the structural level of the CJ (Figs. 4e and 5j, k). Both aspects are playing hand in hand, to fine-tune mitochondrial Ca^2+^ and structural integrity to avoid matrix Ca^2+^ deprivation or uncontrolled H^+^ uncoupling through the CJ^19^. These findings have a profound influence on the understanding of mitochondrial metabolic regulation and how Ca^2+^ signals are decoded into cellular responses.

## Methods

### Structured illumination microscopy (SIM)

#### Single and dual camera SIM imaging

The SIM setup used is composed of a 405, 488, 515, 532, and a 561 nm excitation laser introduced at the back focal plane inside the SIM box with a multimodal optical fiber. For super-resolution, a CFI SR Apochromat TIRF ×100-oil (NA 1.49) objective was mounted on a Nikon-Structured Illumination Microscopy (N-SIM®, Nikon, Austria) System with standard wide field and SIM filter sets and equipped with two Andor iXon3® EMCCD cameras mounted to a Two Camera Imaging Adapter (Nikon Austria, Vienna, Austria). At the bottom port, a third CCD camera (CoolSNAP HQ2, Photometrics, Tucson, USA) is mounted for wide-field imaging. For calibration and reconstruction of SIM images, the Nikon software (NIS-Elements, Nikon, Austria) was used. Reconstruction was permanently performed with the same robust setting to avoid artifact generation and ensures reproducibility with a small loss of resolution of 10% compared to the most sensitive and rigorous reconstruction settings. Microscopy setup adjustments were done as described elsewhere^2^.

#### Cell culture

HeLa (ATCC-CCL-2.2TM) cells were seeded on 1.5H high precision glass cover slips (Marienfeld-Superior, Lauda-Königshofen, Germany) and cultured in DMEM (D5523, Sigma-Aldrich, Darmstadt, Germany) containing 10% FCS, penicillin (100 U/ml), streptomycin (100 µg/ml) and amphotericin B (1.25 µg/ml) (Gibco™, Thermo Fisher Scientific, Vienna, Austria) in a humidified incubator (37 °C, 5% CO_2_/95% air). The origin of cells was confirmed via STR-profiling by the cell culture facility of the Center of Medical Research (ZMF, Graz, Austria).

#### Transfection procedures

HeLa cells were grown under standard culture conditions until 50% confluence was reached, transfected in DMEM (without FCS and antibiotics) with 1.5 µg/ml plasmids or 100 nM siRNA using 2.5 µg/ml TransFast™ transfection reagent (Promega, Madison, WI, USA). After 24 h, the medium was replaced with DMEM containing 10% FCS and 1% penicillin/streptomycin and kept for a further 24 h prior to experiments. The specific siRNAs (Microsynth, Balgach, Switzerland) used in this study are listed in Supplementary Table 1. Alternatively, plasmid transfection was done using PolyJet™ In Vitro DNA Transfection Reagent (SL100688, SignaGen® Laboratories, Frederick, MD, USA).

#### Labeling with MitoTracker™ Green FM and tetramethylrhodamine methyl ester

Cells were washed once with loading buffer containing in mM: 2 CaCl_2_, 135 NaCl, 5 KCl, 1 MgCl_2_, 1 HEPES, 2.6 NaHCO_3_, 0.44 KH_2_PO_4_, 0.34 Na_2_HPO_4_, 10 d-glucose (Carl Roth, Karlsruhe, Germany), 0.1% vitamins, 0.2% essential amino acids and 1% penicillin/streptomycin at pH 7.4. Cells were incubated in loading buffer containing 1000, 500, 200, 81, 40.5, 13.5, 5.4, 2.7, or 1.35 nM TMRM (tetramethylrhodamine methyl ester, Invitrogen™) for 30 min. As TMRM might degrade over time in storage, TMRM concentrations were measured regularly. Therefore, after TMRM was dissolved in methanol the absorption at 550 nm was measured and the concentration was calculated using Eq. 1.

1 $$c=A/\left(\varepsilon \cdot l\right)$$

*c* is the molar concentration of TMRM, *A* the absorption of TMRM at 550 nm, *ε* is the specific extinction coefficient of TMRM at 550 nm in methanol, and *l* is the pathlength the light has to path through the TMRM solution.

### Determination of TMRM quenching at various concentration

HeLa cells seeded on glass slides were labeled for 30 min with 1000, 500, 200, 81, 13.5, or 1.35 nM TMRM and transferred into a perfusion chamber. During the measurements cells were continuously perfused using a gravity-based perfusion system (NGFI, Graz, Austria) Measurements were performed on an inverted wide-field microscope (Observer.A1, Carl Zeiss GmbH, Vienna, Austria) as described previously^15^. TMRM was excited at 550 nm, and the emission was collected at 600 nm. A full disruption of the mitochondrial membrane potential was done by the application of carbonyl cyanide-p-trifluoromethoxyphenylhydrazone (FCCP) (Abcam, Cambridge, UK). In the case of the non-quenching mode of TMRM, a direct drop of mitochondrial fluorescence followed FCCP treatment. In the case of quenching mode, a clear increase in TMRM fluorescence after FCCP addition was observed, followed by a drop in intensity. During the entire experiment the respective TMRM concentrations were present within the imaging buffers and prior recording the cells were equilibrated in the perfusion chamber for a further 5 min. Mitochondrial TMRM signals of single cells were background corrected using a background ROI.

### Determination of TMRM saturation at various concentration

HeLa cells seeded on glass slides were labeled for 30 min with 81, 40.5, 13.5, 5.4, 2.7, or 1.35 nM TMRM and transferred into a perfusion chamber containing the same concentration of TMRM. Per coverslip, 40 cells were imaged randomly and the average cellular TMRM fluorescence was measured.

#### Cristae membrane and inner boundary membrane potential separation by FWHM

HeLa cells transfected with mt-sfGFP were stained with different concentration of TMRM ranging from 1.35 to 81 nM. After the staining procedure the cells were kept in loading buffer containing the respective concentrations of TMRM and imaged with dual-color 3D-SIM. To compensate for intensity differences for TMRM acquisition the laser power was adjusted to match image intensity histograms of different TMRM concentrations. Post imaging, the recorded data were background corrected using an ImageJ Plugin (Mosaic Suite, background subtractor, NIH) with a sliding rectangle diameter of 50 pixel. Intensity line plots of mitochondrial mt-sfGPF and TMRM fluorescence were manually measured with a width of 50 pixels (1.6 µm). The FWHM of mt-sfGFP and TMRM fluorescence distributions was measured using linear interpolation to gain subpixel information. Subtraction of FWHM_mt-sfGFP_ from FWHM_TMRM_ results in ∆FWHM.

#### Cristae membrane and inner boundary membrane potential separation by IBM association index

The IBM association factor of TMRM or MTG was calculated as described elsewhere^2^. In short, images were subjected to background subtraction (Mosaic Suite, background substractor, NIH) with a sliding rectangle diameter of 50 pixels. The reference channel (sf-GFP, mtDsRed, or MTG) was Otsu^45^ auto thresholded and further dilated and eroded in two independent subsets. One erosion and two dilation iterations were used. Pixel-wise subtraction of the erosion reference of the dilated reference image yields a hollow structure, used as a mask to measure the mean intensity in the mitochondrial periphery or IBM-related area in the object channel. The erosion reference served as a mask to measure the bulk or cristae mean fluorescence intensity. The ratio of IBM/CM mean intensity is a value to estimate changes in the object label distribution inside a mitochondrion, which is referred to as the IBM association index. The higher the ratio value the higher the distribution of protein labels in the IBM. For image analysis the freeware program ImageJ was used.

#### Morphological analysis of mitochondria in 2D-SIM images

3D-SIM and time-lapsed images of EMRE-mCherry, MTR, MTG, or MICU1-YFP were used for morphological analysis. Images were background corrected with an ImageJ Plugin (Mosaic Suite, background subtractor, NIH) and the subsequent binarization was done using an Otsu^45^ auto threshold. The ImageJ particle analyzer was used to extract the mitochondrial count (*c*), area (*a*), perimeter (*p*), minor (*x*), and major (*y*) axes of the mitochondria. Aspect ratio (AR) was determined as

2 $${{{{{{\rm{AR}}}}}}}=\frac{y}{x}$$

The Form Factor (FF) was determined as followed:

3 $${{{{{{\rm{FF}}}}}}}=\frac{{p}^{2}}{4\pi \cdot a}$$

#### Electron microscopy

Cells were incubated in a loading buffer with 81, 13.5, or 1.35 nM TMRM for 30 min, washed with PBS, fixed with 2.5% glutardialdehyde and 2% formaldehyde in a buffered solution, and postfixed in either 2% osmium tetroxide or 1% osmium tetroxide that had been reduced with 1% potassium hexacyanoferrate^46^. The cells were dehydrated in an ascending ethanol series, embedded in TAAB embedding resin, and sectioned on a Leica Ultracut 7 ultramicrotome using a Diatome diamond knife. The sections were counter-stained using platinum blue (IBIlabs, Boca Raton, FL, USA) and lead citrate (Leica Microsystems, Wetzlar, Germany) and visualized in an FEI Tecnai 20 transmission electron microscope. They were photographed at ×2000 magnification with a Gatan ultrascan 1000 camera.

#### Analysis of cristae membrane and density

In a first step, mitochondria were segmented inside the micrographs manually by free hand selection. Afterwards, the segmented mitochondria were isolated and the cristae inside each mitochondrion was segmented by free hand selection in ImageJ. The process was semi-automated using ImageJ macros. Mitochondrial and cristae area and perimeter were measured. The perimeter of mitochondria divided by the cristae perimeter gives an indication of membrane alteration of the cristae. The ratio of cristae perimeter divided by mitochondrial area gives a representation of cristae density.

#### Analysis of cristae density distribution

For analysis of the spatial cristae density distribution, segmented mitochondria and respective cristae were selected. Segmented mitochondria were binarized and used as a mask for binarized cristae membranes to determine the percentage coverage of cristae perimeter inside the mitochondria area. Iteratively, the mitochondrial mask was eroded homogeneously in small increments of 2-pixel width (5.88 nm) and the respective coverage of cristae was measured. The result of these measurements represented a cristae density in circular segments starting from the outer mitochondrial membrane towards the mitochondrial center. As mitochondrial size and shape vary that results in different numbers of ring segments, the cristae densities were normalized by linear interpolation to 100 segments.

#### Analysis of local drops in membrane potential in close proximity to MAMs

Cells were transfected with an insensitive ER targeted ATP probe ERAT4.03 NA (NGFI, Graz, Austria^47,48^) as a marker for the ER and stained with 13.5 nM TMRM in loading buffer for 30 min. TMRM was present during the entire experiment. Cells were afterwards transferred to the live cell chamber containing 0.8 ml loading buffer and imaged over time with a frequency of 0.25 Hz. 8 s after image sequence initialization 0.8 ml of loading buffer with or without 200 µM histamine was added to a final concentration of 100 µM histamine.

To analyze whether local drops of membrane potential appear under SOCE, cells were incubated with 2 µM Fura-2AM (MoBiTec GmbH, Göttingen, Germany) and 13.5 nM TMRM in loading buffer for 30 min. TMRM was present during the entire experiment. Cells were challenged in Ca^2+^-free buffer with 100 µM histamine/10 µM CPA (Sigma-Aldrich, Vienna, Austria) to empty ER-Ca^2+^ stores and imaged using the widefield setup with 380 nm illumination and widefield acquisition with the bottom port CCD camera. Afterward, the same cells were imaged over time with a frequency of 0.25 Hz using the dual-cam SIM setup. 8 s after image sequence initialization Ca^2+^ was added to a final concentration of 2 mM.

A custom-made ImageJ macro was used to analyze spatial drops in mitochondrial membrane potential in dependency of spatial proximity to ER-related mitochondrial-associated membranes (MAM) and ER Ca^2+^ release. The analysis was separated into several steps, (1) definition of MAMs, (2) identification of drops in the mitochondrial membrane potential, and (3) spatial assignment of the drops to MAM and non-MAM mitochondrial areas.

1. After acquisition and reconstruction using NIS-Elements AR the mitochondrial TMRM channel (further referred to as C_M_) and the ERAT4.03 NA channel (further referred to as C_E_) were background subtracted (Mosaic Suite, background subtractor, NIH) with a sliding rectangle diameter of 50 pixels. Both channels (C_M_ and C_E_) were bleaching corrected using the histogram matching function, which is implemented in Fiji. An Otsu auto threshold was used to binarize C_M_ and C_E_. The overlap of both channels was eroded (iteration = 1; count = 1) to remove small structures and subsequently dilated (iteration = 6; count = 1) and used as a mask for C_M_ to assign areas of mitochondria with close proximity to MAMs.
2. Background subtracted (Mosaic Suite, background subtractor, 50 pixels, NIH) and bleaching corrected (histogram matching) C_M_ was analyzed for frame-to-frame intensity changes (∆C_M_). Additionally, Otsu auto thresholded C_M_ of both images involved for the calculation of ∆C_M_ was used as a mask for ∆C_M_ to remove the mitochondrial movement from ∆C_M_. Negative changes from one frame to the next (−∆C_M_) as well as positive ones (+∆C_M_) were subjected to a threshold of 35% of the original intensity of the first frame. Thresholded −∆C_M_ and +∆C_M_ were eroded (iteration = 1; count = 1) and subsequently dilated (iteration = 1; count = 1) to remove remaining halos around mitochondria still originating form mitochondrial movement. Finally, the area of −∆C_M_ and +∆C_M_ containing segments were quantified and the ratio of −∆C_M_/+∆C_M_ was calculated. For more easy interpretation, −∆C_M_ and +∆C_M_ were replaced with −∆TMRM and +∆TMRM, respectively. The area of −∆C_M_ quantifies distinct local losses of TMRM signal, which can be induced by direct loss of membrane potential or by the movement of the cristae membrane itself. Shifts in the lateral or axial direction within mitochondria can lead to a loss of TMRM fluorescence. Nevertheless, cristae membrane movement should account for increases in TMRM signals in the same quantity. A ratio of −∆/+∆TMRM = 1 states that equal amounts of distinct changes, increase or loss of TMRM signal, are present and can be referred to a steady state in which movement of cristae membrane is the main contribution to −∆TMRM and +∆TMRM. −∆/+∆TMRM < 1 shows more increases in TMRM signal indicating the generation of mitochondrial membrane potential. −∆ /+∆TMRM > 1 on the other side implies a local high loss of membrane potential.

The whole mitochondrial area derived from auto Otsu binarized CM as well as MAMs determined in (1) were used as a mask for −∆TMRM and +∆TMRM areas to differentiate −∆/+∆TMRM-ratios in regard to the proximity to MAMs.

#### Technical note

Lateral and axial movements of the mitochondria and the CM over time may cause errors in the readings of the intensiometric TMRM signals. To counter this issue, the delta TMRM fluorescence (∆C_M_) of subsequent frames was calculated and thresholded by ± 35% of the fluorescence intensity change per frame and per pixel and classified them into positive (+∆TMRM) and negative (−∆TMRM) area partitions. Accordingly, the ratio of −∆/+∆TMRM compensates for errors due to CM movements and quantifies local variations of membrane potential of the IMM. The ER-marker was used to identify mitochondrial structures in close proximity to the ER (MAMs) (Fig. 4a). The ratio of −∆/+∆TMRM was calculated over time.

### Ca^2+^ imaging experiments

Ca^2+^ imaging was performed as described elsewhere^49^. In brief, Ca^2+^ was measured on a digital wide-field microscope, the iMIC (Till Photonics, Gräfelfing, Germany) equipped with a 40-objective (alpha Plan Fluar 40, Zeiss, Göttingen, Germany) and an ultrafast switching monochromator, the Polychrome V (Till Photonics). Illumination of GEM-GeCO1 targeted sensors was performed at 430 nm excitation and emissions were collected with a dichrotome dual emission filter set (dichroic 535dcxr). For dual recordings, GEM- and CAR-GeCO1 targeted sensors were alternately excited for 500 ms each at 430 and 575 nm. Emissions derived from both sensors were taken in 3 s intervals. During the measurements, cells were continuously perfused using a gravity-based perfusion system (NGFI, Graz, Austria) and images were recorded with a charge-coupled device (CCD) camera (AVT Stingray F-145B, Allied Vision Technologies, Stadtroda, Germany). Data acquisition and control of the digital fluorescence microscope were performed using the live acquisition software version 2.0.0.12 (Till Photonics). For analysis, fluorescent traces of single cells were background corrected using a background ROI, and bleaching was corrected using an exponential decay function in a costume made excel macro. To compare the kinetics of two mitochondrial compartments, the fluorescent traces were normalized to the minimum and maximum of the IP_3_ or SOCE-induced mitochondrial Ca^2+^ elevations. The area under the curve was determined for both compartments using the trapezium method. The delta of both AUCs was calculated. The starting point of the ∆AUC was set by the point at which the Ca^2+^ signal in one of both compartments surpasses the three-fold of the standard deviation of the basal signal. The end of the ∆AUC was set to the time point at which one of the signals reached the maximum and crosses with the intensity of the opposite Ca^2+^ trace from the other sub-mitochondrial compartment.

### Western blot

Western blots were performed according to standard protocols. Briefly, cell lysis was conducted with RIPA buffer (Bio-Rad formulation) supplemented with protease inhibitor cocktail (1:50; Sigma Aldrich, Vienna, Austria), followed by sonication (80% amplitude, 2 × 15 s). Samples were denatured in 1× Laemmli sample buffer and resolved on a 7.5 or 12.5% SDS-PAGE gel together with PageRuler™ Plus Prestained Protein Ladder (Fisher Scientific, Vienna Austria). Blots were blocked and antibodies diluted in 5% BSA (Sigma Aldrich) in TBS-T. The following antibodies were used: MICU1 (D4P8Q, 1:1000, Cell Signaling Technology, MA, USA), and Histone H3 (1B1B2, 1:1000, Cell Signaling Technology). HRP labeled anti-mouse (PI-2000, 1:1000, Vector Laboratories, Burlingame, USA) and anti-rabbit (sc-2357, 1:1000, Santa Cruz Biotechnologies) were used as secondary antibodies. For visualization, the SuperSignal West Pico PLUS kit (Fisher Scientific) was used and detection was conducted on the ChemiDoc System (Bio-Rad Laboratories, Vienna, Austria).

### Statistics and reproducibility

Each exact *n* value and the number of independent experiments are indicated in each figure legend. Statistical analysis was performed using the GraphPad Prism software version 5.04 (GraphPad Software, San Diego, CA, USA) or Microsoft Excel (Microsoft Office 2013). Analysis of variance (ANOVA) with Bonferroni post hoc test and *t* test were used for evaluation of the statistical significance. All box plots show minimum to maximum values if not otherwise indicated. The central line is the median with boxes extending to 25 and 75% and the whiskers encompass all data. *P* < 0.05 was defined to be significant. At least three experiments on 3 different days were performed for each experimental set-up.

### Reporting summary

Further information on research design is available in the Nature Research Reporting Summary linked to this article.

## Supplementary information

Peer Review File

Supplemental Material

Description of Additional Supplementary Files

Supplementary Data

Reporting Summary

## Data Availability

The data that support the findings of this study are available from the authors on reasonable request, see author contributions for specific data sets.

## Code availability

The code used in this study is available from the authors on reasonable request; see “Author contributions” for specific code sets.
