# Supplementary material for: MICU1 controls spatial membrane potential gradients and guides Ca2+ fluxes within mitochondrial substructures
Source: Commun Biol. 2022 Jul 1;5:649. doi: 10.1038/s42003-022-03606-3 (PMC9249747; doi:10.1038/s42003-022-03606-3)
Supplement: Supplementary file 2 — Supplemental Material [file 42003_2022_3606_MOESM2_ESM.pdf]

Supplementary figure 1:

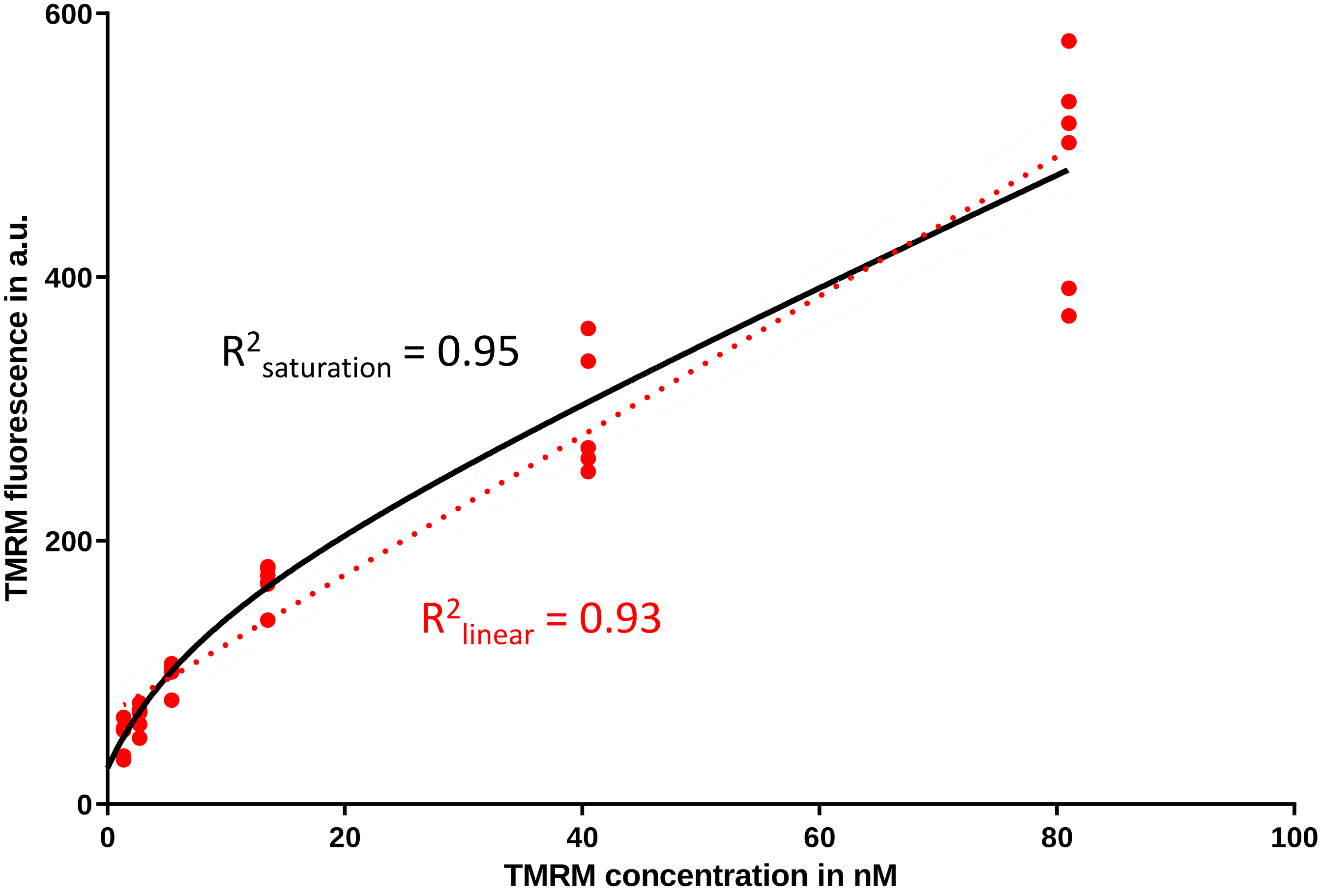

Supplementary Figure 1: Representative curves of HeLa cells, stained with 1000, 500, 200, 81, 13.5, or 1.35 nM tetramethylrhodamine methyl ester (TMRM) (magenta) for 30 min. 40 Cells were imaged with identical imaging setup per glass cover slip and the average TMRM intensity per cell was measured. The experiment was repeated twice on 3 different days (n=6). Both, a linerar and saturation kinetic fitting were applied. The respective R^2–values are displayed in the graph. Source data are provided as a Source Data file.

Supplementary figure 2:

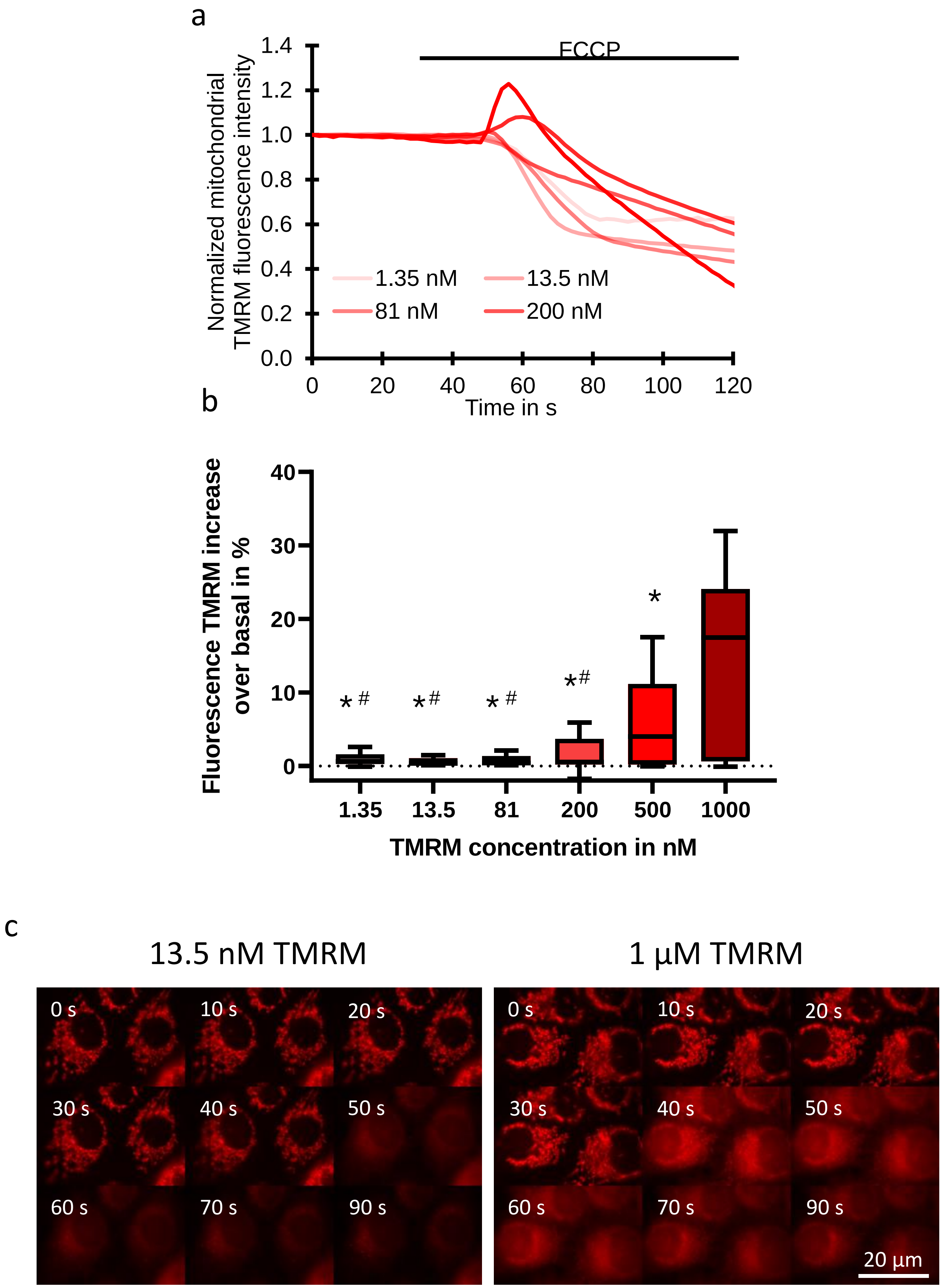

Supplementary Figure 2: **a** Representative curves of HeLa cells, stained with 1000, 500, 200, 81, 13.5, or 1.35 nM tetramethylrhodamine methyl ester (TMRM) (magenta) for 30 min. The cells were treated with FCCP to evaluate the quenching state of TMRM at different concentrations. **b** The maximal delta of basal fluorescence and the sharp fluorescence increase after FCCP addition was quantified as a measure for the quenching state of TMRM. Horizontal lines in **b** represent the median, the lower and upper hinge show respectively first quartile and third quartile, and lower and upper whisker encompass 10% and 90% values. **c** Representative montages of HeLa cells labeled with 13.5 nM or 1µM TMRM and treated with FCCP according to the protocol shown in (a). The experiment was repeated 4 times including a total of 60 cells per condition N = 60. * $p<0.05$ to 1000nM # $p<0.05$ to 500nM respective indicated conditions carried out with 1-way analysis of variance (ANOVA) with Bonferroni post hoc test. Source data are provided as a Source Data file.

Supplementary figure 3:

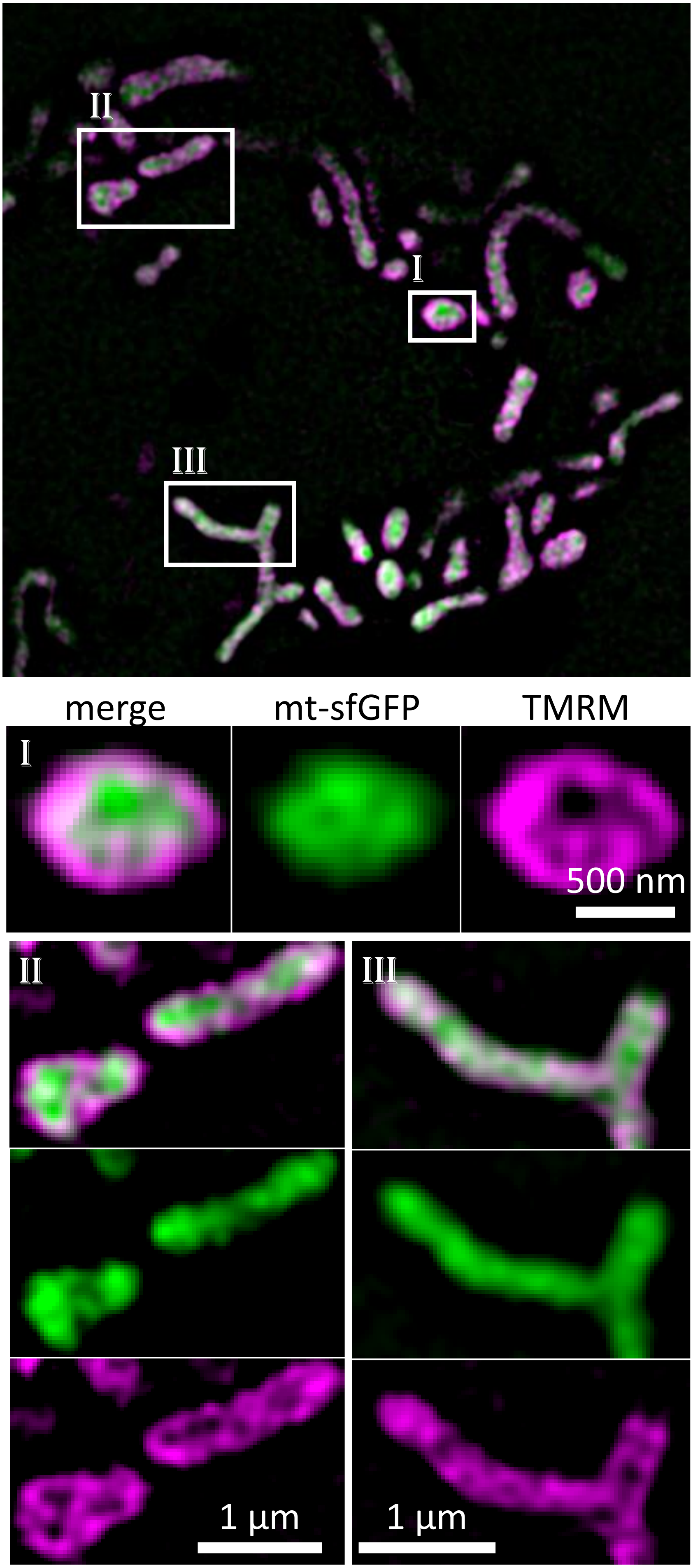

Supplementary Figure 3: TMRM accumulates in the inner mitochondrial membrane. Hela cells expressing mitochondrial matrix targeted sfGFP were incubated for 30 min in 81 nM TMRM treated with ATP to induce mitochondrial swelling and imaged with SIM microscopy. In (I) swollen mitochondria an exclusive central matrix staining without TMRM can be seen. Elongated mitochondria (II & III) show cristae like TMRM staining pattern surrounding the central matrix targeted mt-sfGFP signal.

Supplementary figure 4:

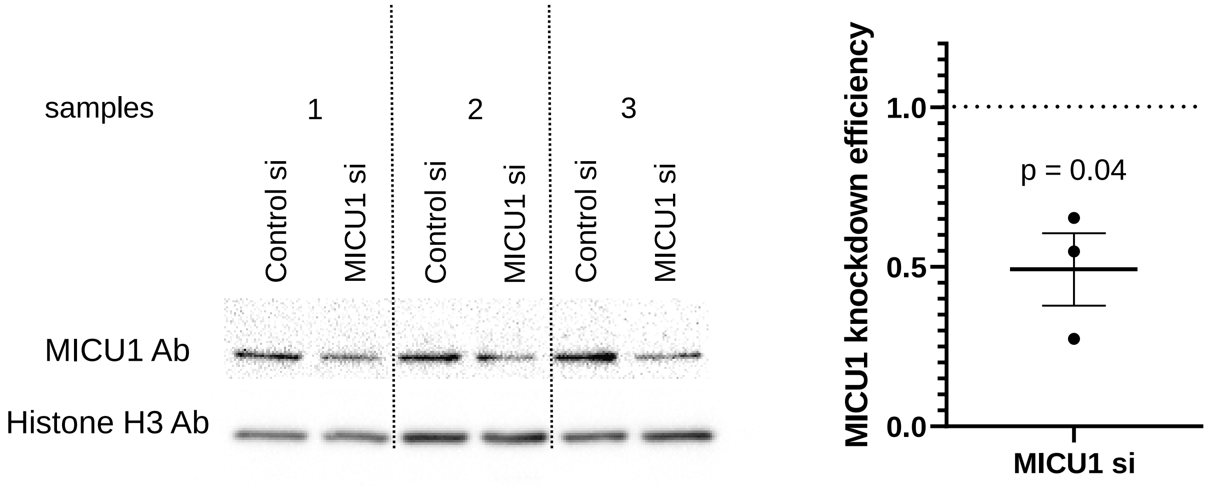

Supplementary Figure 4: Quantification of knockdown efficiency of MICU1 by Western blot. Verification of knockdown efficiency of MICU1 after treatment with scrambled control siRNA or siRNAs against MICU1. (Left panels) Representative Western blot and (right panels) corresponding statistical analysis of Western blots. For Western blot Histone H3 was used as a loading control . Data are shown as dot plots with the mean +/- SEM as middle line and whiskers, respectively (n=3). Source data are provided as a Source Data file.

Supplementary figure 5:

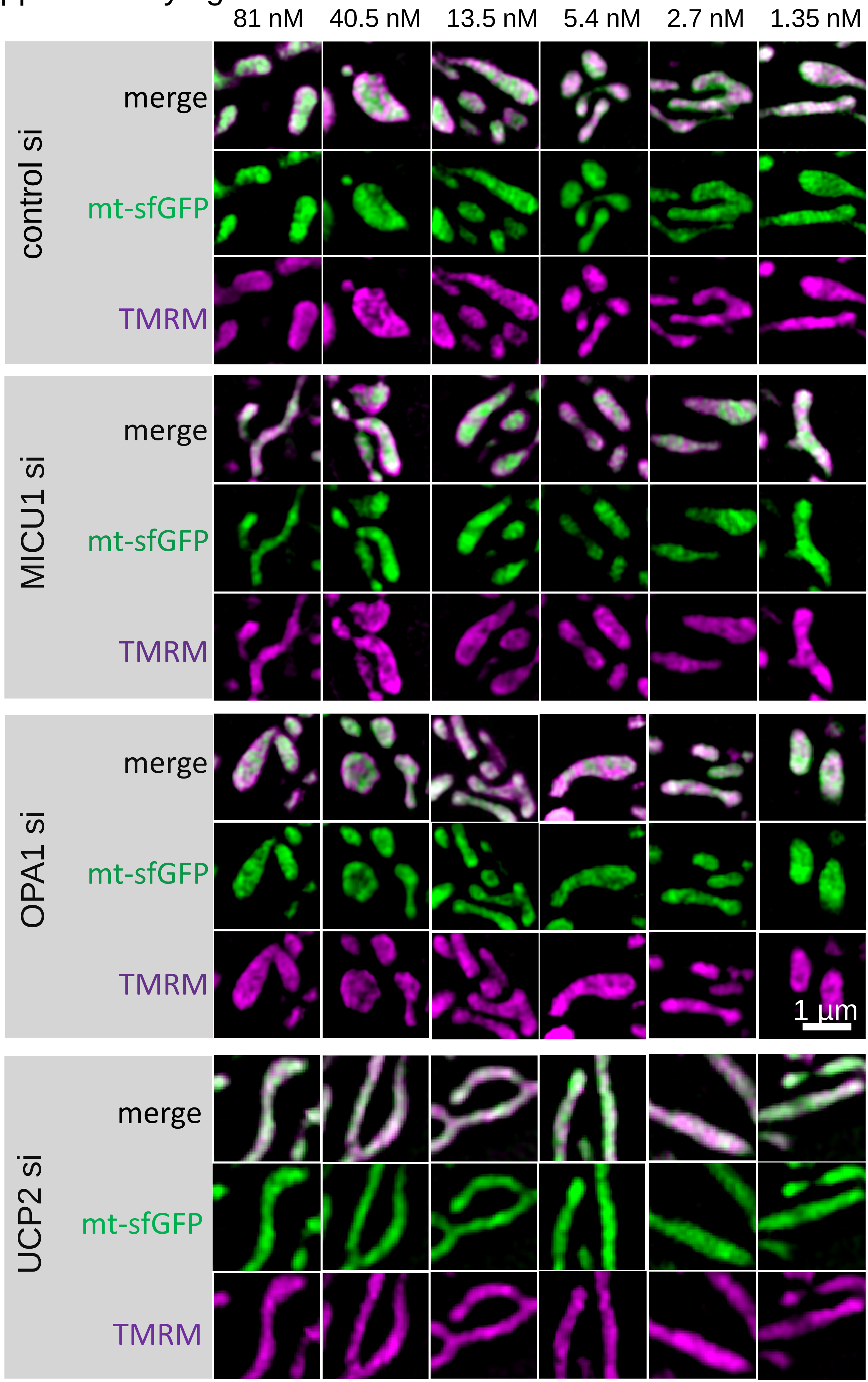

Supplementary Figure 5: Representative images of HeLa cells expressing mt-sfGFP (green), stained with 81, 40.5, 13.5, 5.4, 2.7, or 1.35 nM tetramethylrhodamine methyl ester (TMRM) (magenta) and examined using simultaneous dual-color 3D-SIM as shown in Figure 3a. Overlays and single channel images are shown for control si, MICU1 si, OPA1 si and UCP2 si transfected cells.

Supplementary figure 6:

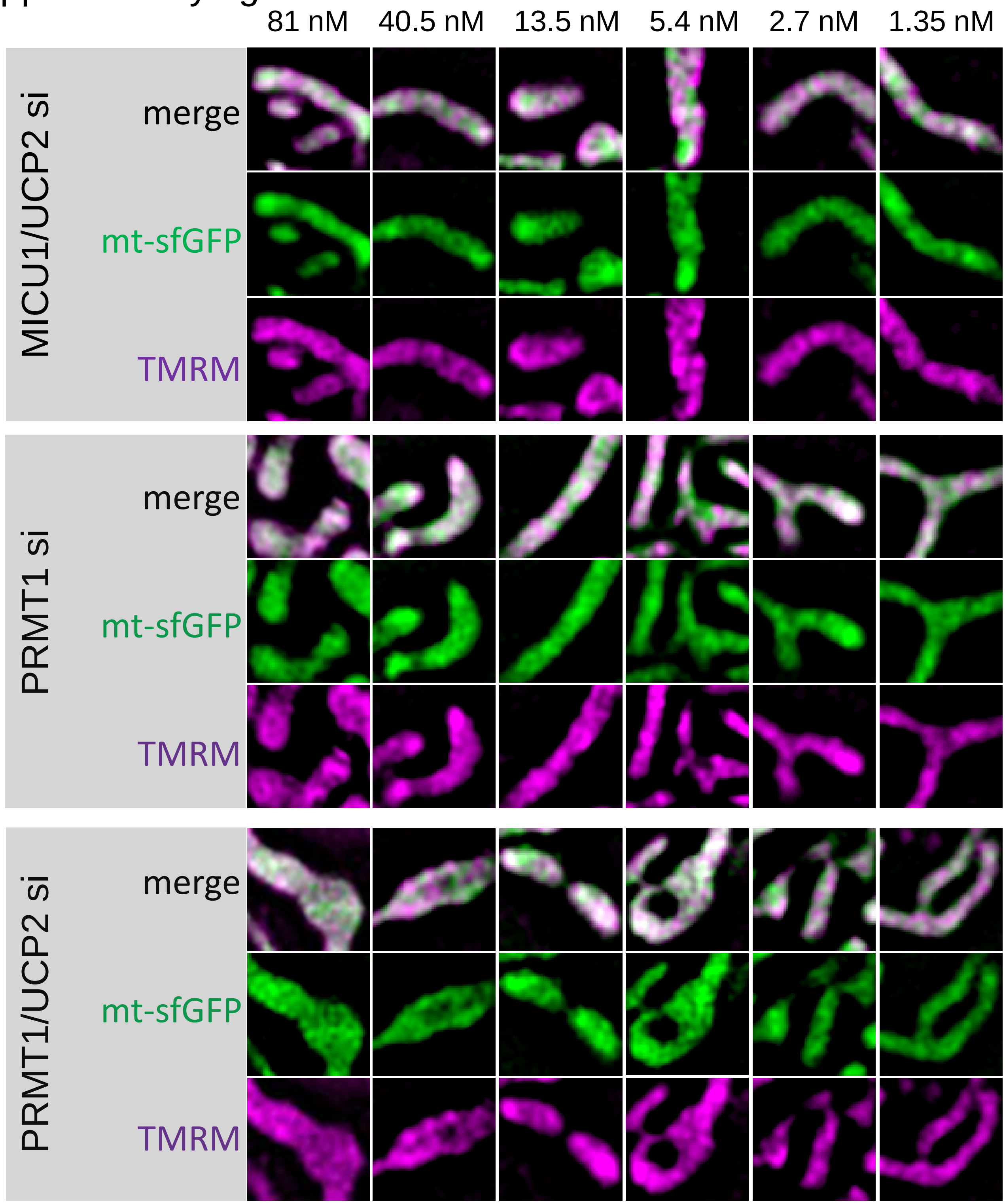

Supplementary Figure 6: Representative images of HeLa cells expressing mt-sfGFP (green), stained with 81, 40.5, 13.5, 5.4, 2.7, or 1.35 nM tetramethylrhodamine methyl ester (TMRM) (magenta) and examined using simultaneous dual-color 3D-SIM as shown in Figure 3a. Overlays and single channel images are shown for MICU1/UCP2 si, PRMT1 si, and PRMT1/UCP2 si transfected cells.

Supplementary figure 7:

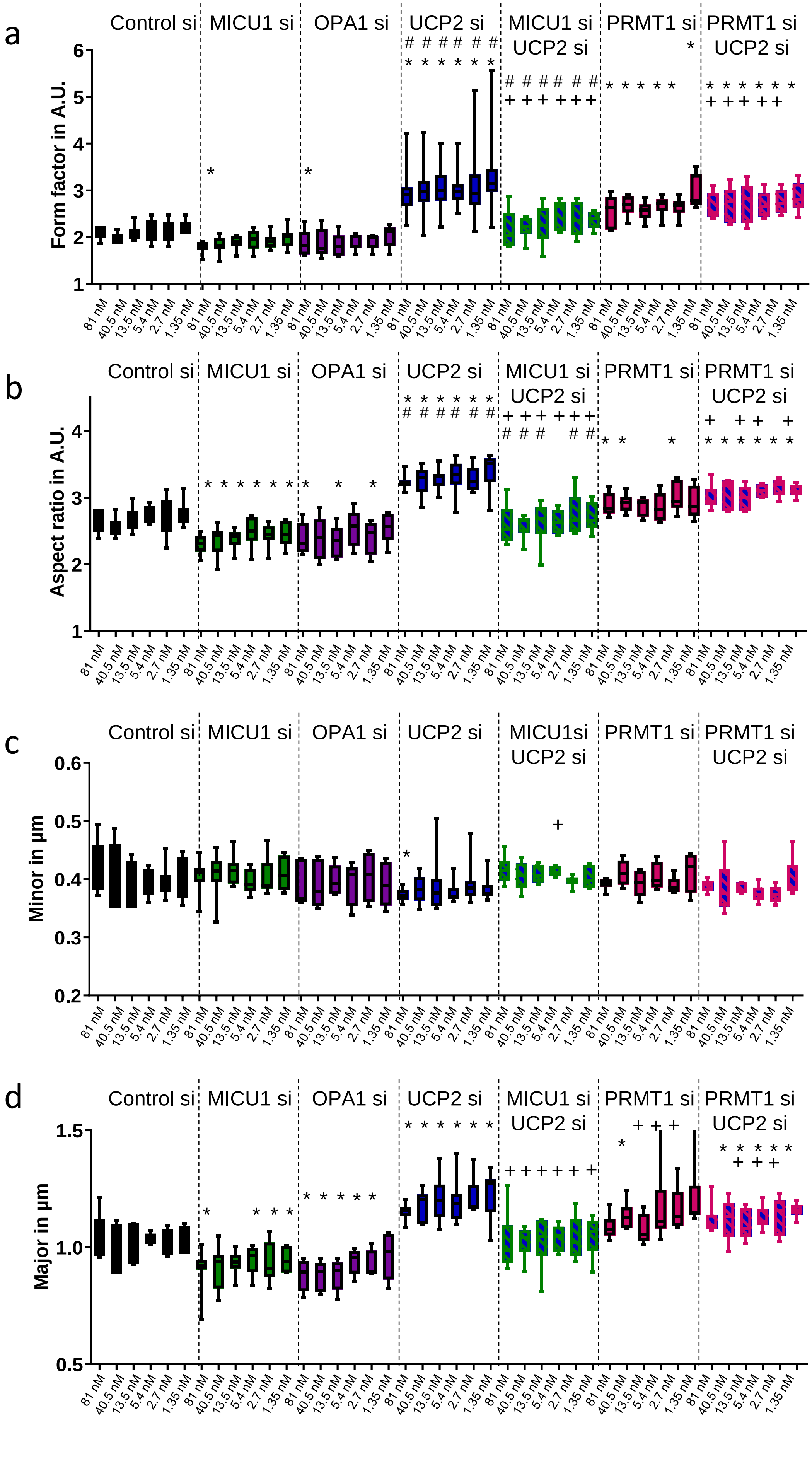

Supplementary Figure 7: Influence of MICU1, OPA1 UCP2 and PRMT1 on mitochondrial morphology. HeLa cells expressing mt-sfGFP and transfected with control, MICU1, OPA1, PRMT1 or UCP2 siRNA were stained with 81, 40.5, 13.5, 5.4, 2.7, or 1.35 nM TMRM. The mitochondrial morphology indicators from factor **a**, aspect ratio **b**, as well as shape descriptors minor **c** and major **d** diameter were calculated. Horizontal lines represent the median, the lower and upper hinge show respectively first quartile and third quartile, and lower and upper whisker encompass 10% and 90% values. Images and analyses were obtained from each 10 cells in 3 - 7 independent experimental days ($n_{\text{Control si}} = 8$, $n_{\text{MICU1 si}} = 7$, $n_{\text{OPA1 si}} = 5$, $n_{\text{UCP2 si}} = 10$, $n_{\mathit{MICU1}/\text{UCP2 si}} = 6$, $n_{\text{PRMT1 si}} = 6$, $n_{\mathit{PRMT1/U}\text{CP2 si}} = 6$). * $P < 0.05$ vs. respective control and # $P < 0.05$ vs. respective UCP2 si conditions carried out with 2-way analysis of variance (ANOVA) with Bonferroni post hoc test. Source data are provided as a Source Data file.

Supplementary figure 8:

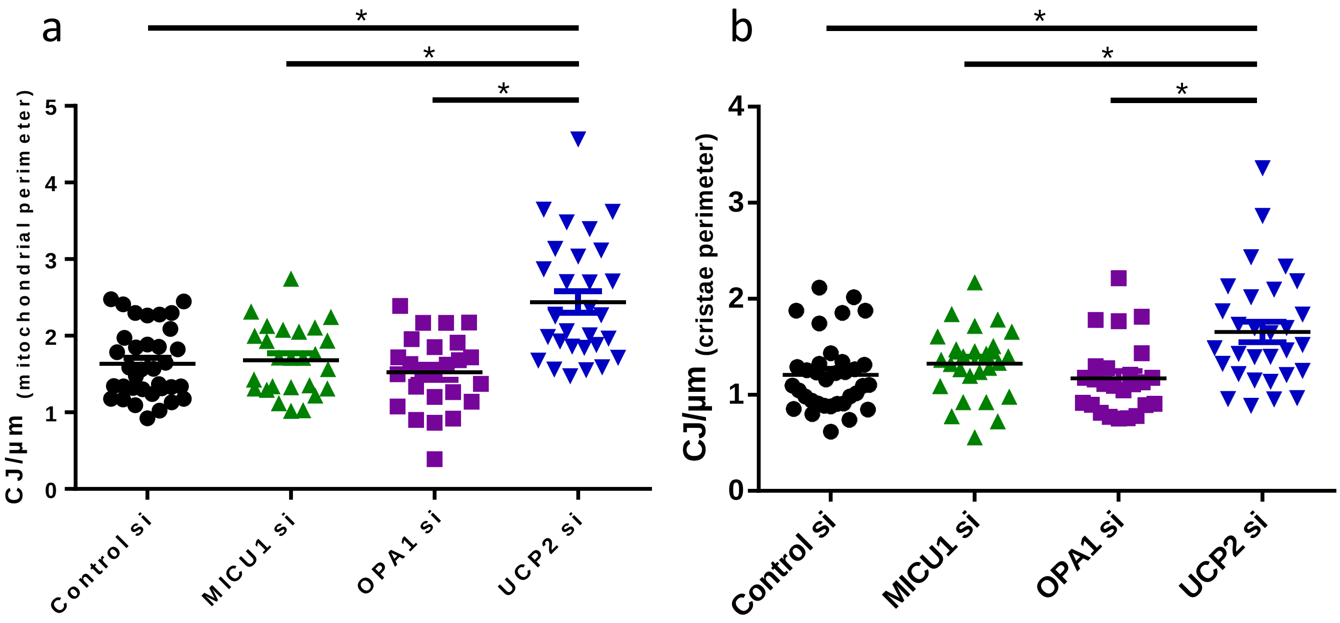

Supplementary Figure 8:UCP2 increases number of CJ **a** CJ per µm mitochondrial perimeter and **b** CJ per µm cristae perimeter were analyzed by counting manually the CJ for each mitochondrion and normalizing the count to the individual perimeter of the mitochondrion or the cristae within the mitochondrion. Data are shown as dot plots with the mean +/- SEM as middle line and whiskers, respectively ($n_{Control\ si}$ = 35, $n_{MICU1\ si}$ = 26, $n_{OPA1\ si}$ = 25, $n_{UCP2\ si}$ = 30). * $P < 0.05$ vs. respective indicated conditions carried out with 1-way analysis of variance (ANOVA) with Bonferroni post hoc test. Source data are provided as a Source Data file.

Supplementary figure 9:

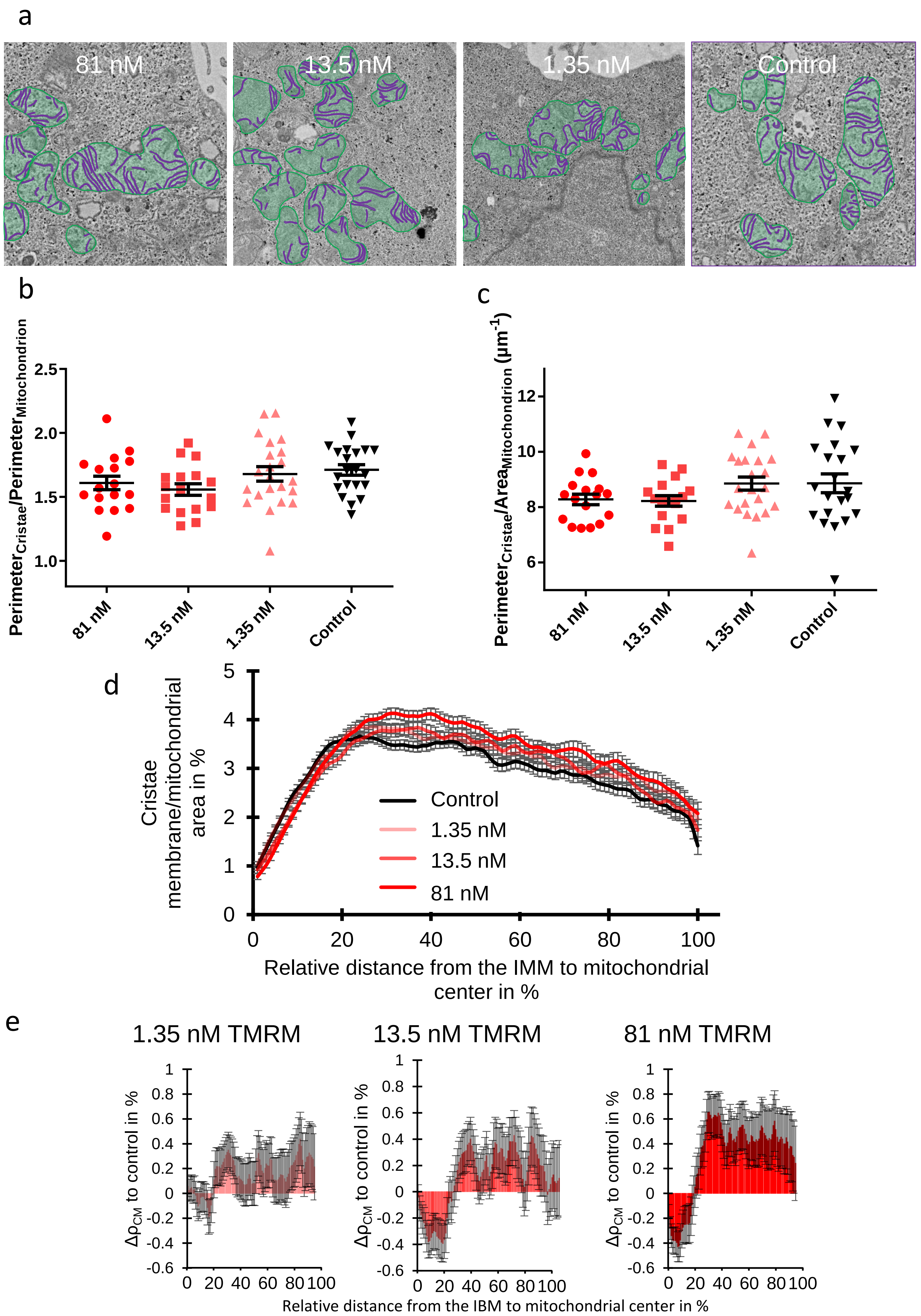

Supplementary Figure 9: TMRM does not influence mitochondrial cristae density or distribution. HeLa cells incubated with 81, 13.5 or 1.35 nM TMRM or DMSO (control) were imaged with transmission electron microscopy. **a** Mitochondria and respective cristae were segmented by hand mitochondrial area and perimeter as well as the cristae perimeter were measured. **b**The ratio of cristae to mitochondrial perimeter was measured. Data are shown as dot plots with the mean +/- SEM as middle line and whiskers, respectively. **c** The ratio of cristae perimeter to mitochondrial area was measured to analyze cristae density. Data are shown as dot plots with the mean +/- SEM as middle line and whiskers, respectively. **d** Cristae density was measured in mitochondrial circular segments starting at the IBM interface proceeding to the mitochondrial center. **e** For better illustration the delta cristae density ($\Delta\rho_{CM}$) to control was calculated for 1.35 nM, 13.5 nM and 81 nM TMRM incubated cells. $\Delta\rho_{CM}$ to control cells of 1.35 nM, 13.5 nM and 81 nM TMRM incubated cells were plotted in relation to the relative distance from the IBM. Data are shown as the mean +/- SEM over time ($c_{control}$ = 2/21/187, $n_{1.35\ \mathrm{nM}}$ = 2/22/206, $n_{13.5\ \mathrm{nM}}$ = 2/17/180, $n_{13.5\ \mathrm{nM}}$ = 2/17/200 representing preparations/cells/mitochondria). Source data are provided as a Source Data file.

Supplementary figure 10:

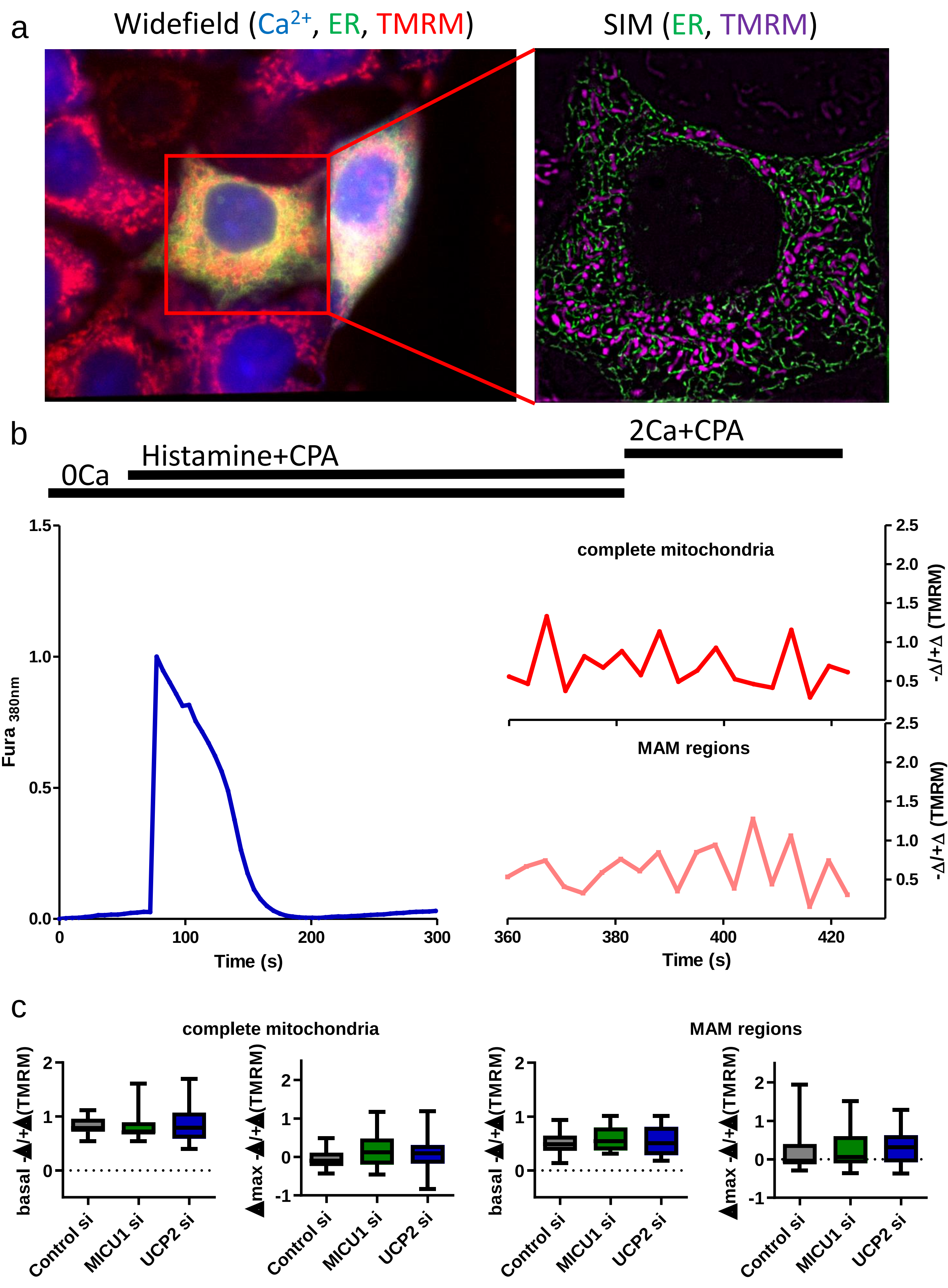

Supplementary Figure 10: SOCE does not induce local drops in $\Delta\Psi_m$. **a** Representative image of ERAT4.01 NA transfected and Fura2-AM, TMRM loaded cells using the widefield bottom port camera (left panel) and the corresponding SIM-image (right panel) **b** Prior to the quantification of local drops of $\Delta\Psi_m$ expressed as -Δ/+ΔTMRM over time in Hela cells (right panel) shown in **a** cells were challenged with 100 µM Histamine/10 µM CPA in Ca^{2+} free buffer to deplete the ER-Ca^{2+} storage (left panel). At T = 380 s CaB was added to stimulate SOCE driven cytosolic Ca^{2+} uptake. **c** Basal and Δmax drops of $\Delta\Psi_m$ expressed as -Δ/+ΔTMRM of complete mitochondria (left panels) and MAM-close regions (right panels) for cells treated with control, MICU1 or UCP2 siRNA. Data are shown as the Boxplot. Horizontal lines in **c** represent the median, the lower and upper hinge show respectively first quartile and third quartile, and lower and upper whisker encompass 10% and 90% values. ($n_{\text{Control si, control}}$ = 4/16, $n_{\text{MICU1 si, control}}$ = 4/15, $n_{\text{UCP2 si, control}}$ = 4/15, with days/cells). *$P < 0.05$ vs. respective Control siRNA conditions carried out with unpaired double-sided T-test. Source data are provided as a Source Data file.

Supplementary figure 11:

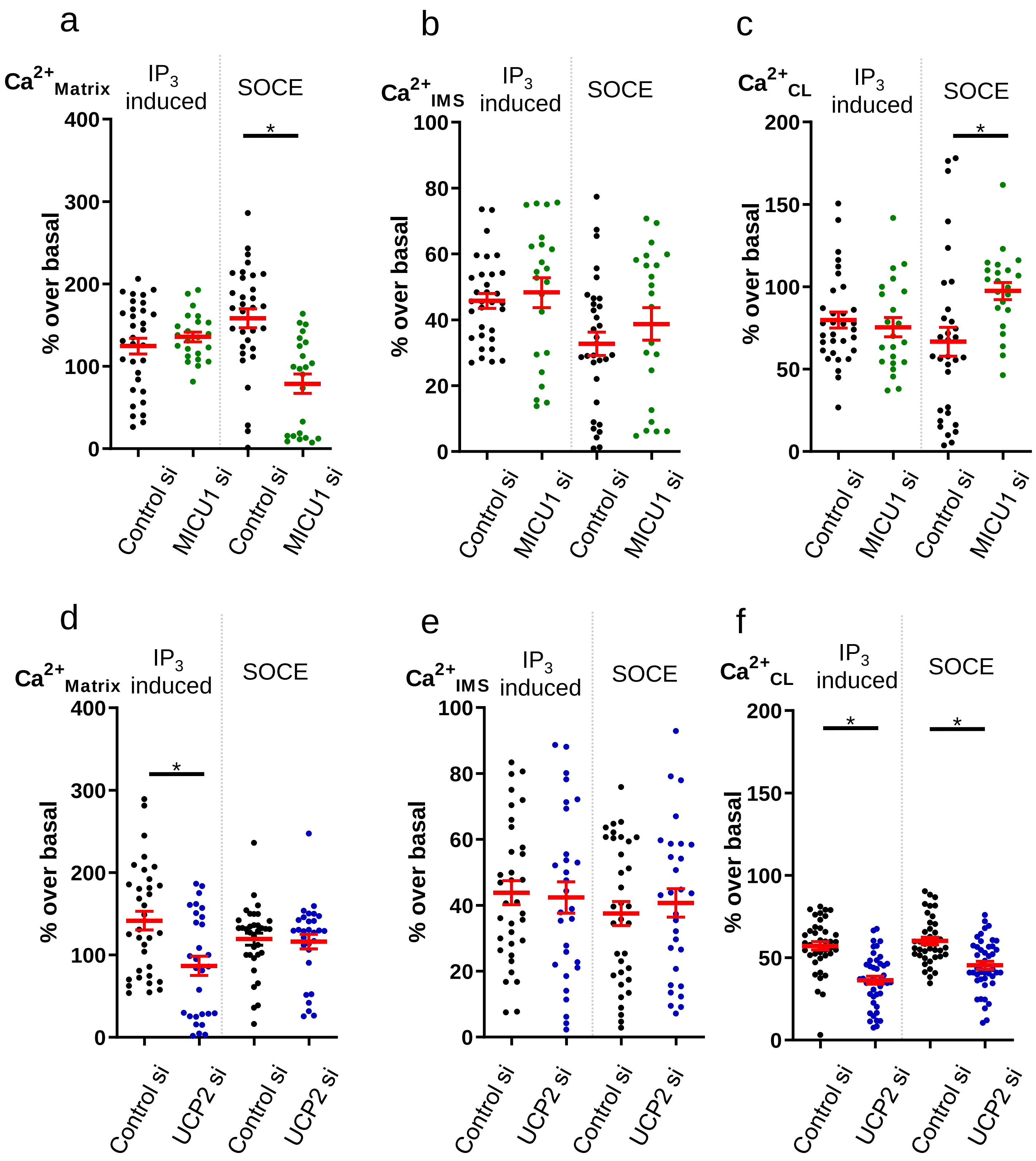

Supplementary Figure 11: MICU1 and UCP2 modulate sub-mitochondrial Ca^{2+} signals under IP_3 and SOCE induced cytosolic Ca^{2+} elevations. Sub-mitochondrial Ca^{2+} concentration under IP_3 or SOCE induced mitochondrial Ca^{2+} uptake was measured in Hela cells treated with control or MICU1 siRNA with **a** mt-CARGeCO1 ($n_{IP3,controlsi}$=32; $n_{IP3,MICU1si}$=23; $n_{SOCE,controlsi}$=32; $n_{SOCE,MICU1si}$=23), **b** IMS-GEMGeCO1 ($n_{IP3,controlsi}$=32; $n_{IP3,MICU1si}$=23; $n_{SOCE,controlsi}$=32; $n_{SOCE,MICU1si}$=23), and **c** CL-GEMGeCO1 ($n_{IP3,controlsi}$=33; $n_{IP3,MICU1si}$=23; $n_{SOCE,controlsi}$=33; $n_{SOCE,MICU1si}$=23). Sub-mitochondrial Ca^{2+} concentration under IP_3 or SOCE induced mitochondrial Ca^{2+} uptake was measured in Hela cells treated with control or UCP2 siRNA with **d** mt-CARGeCO1 ($n_{IP3,controlsi}$=34; $n_{IP3,UCP2si}$=29; $n_{SOCE,controlsi}$=34; $n_{SOCE,UCP2si}$=29), **e** IMS-GEMGeCO1 ($n_{IP3,controlsi}$=34; $n_{IP3,UCP2si}$=29; $n_{SOCE,controlsi}$=34; $n_{SOCE,UCP2si}$=29), and **f** CL-GEMGeCO1 ($n_{IP3,controlsi}$=40; $n_{IP3,UCP2si}$=44; $n_{SOCE,controlsi}$=40; $n_{SOCE,UCP2si}$=44). *$P < 0.05$ vs. respective Control siRNA conditions carried out with unpaired double-sided T-test. Source data are provided as a Source Data file.

Supplementary figure 12:

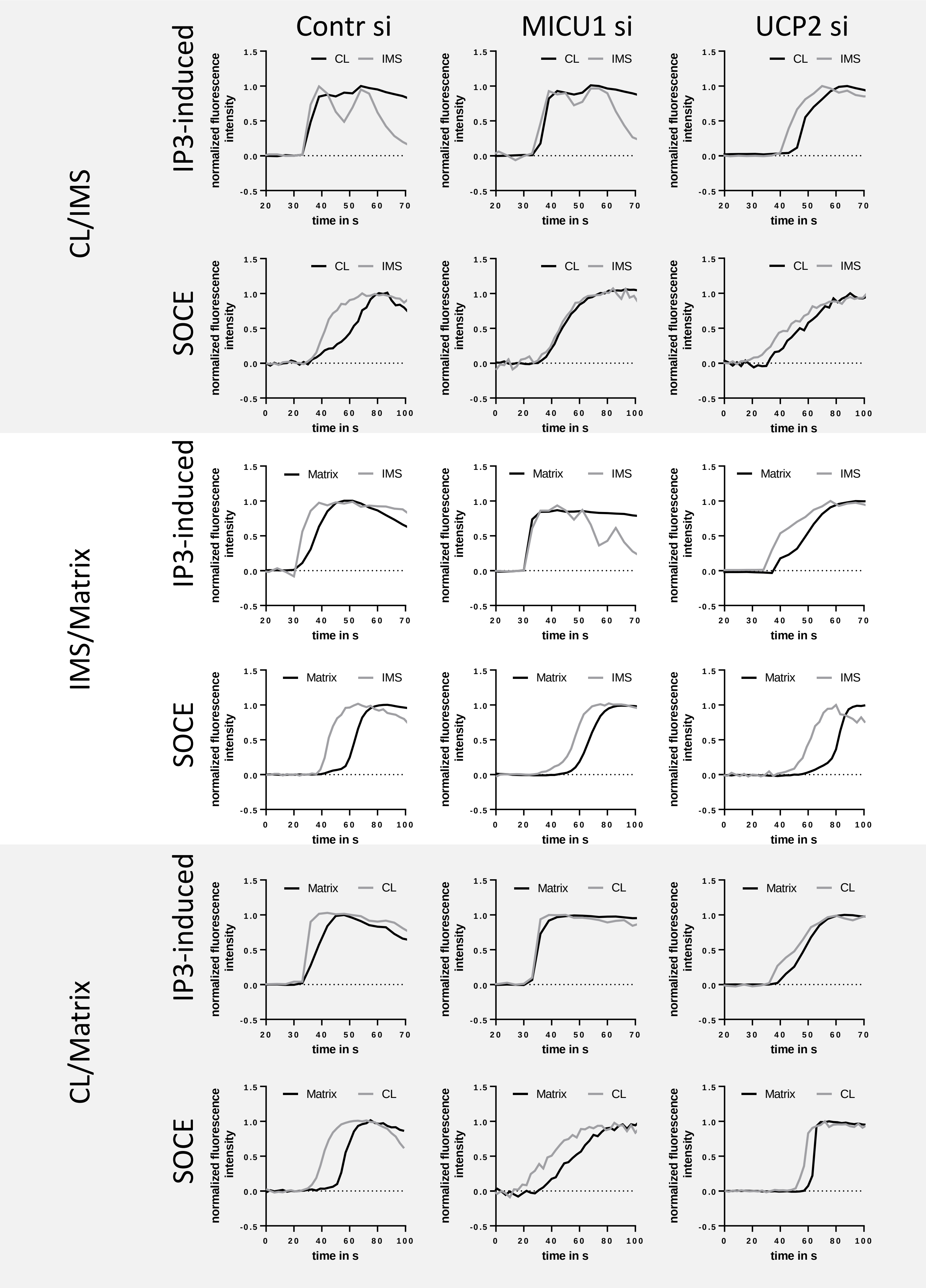

Supplementary Figure 12: Modulation of sub-mitochondrial Ca^{2+} transients by MICU1 and UCP2. Representative sub-mitochondrial Ca^{2+} transients under IP_3 or SOCE induced mitochondrial Ca^{2+} uptake are shown in Hela cells treated with control or MICU1 siRNA with and expressing IMS-GEMGeCO1/mt-CARGeCO1 (IMS/Matrix), IMS-GEMGeCO1/CL-CARGeCO1 (IMS/CL), or CL-GEMGeCO1/mt-CARGeCO1(CL/Matrix) sensor combinations.

Supplementary table 1:

	Target	Sequence
si RNA	MICU1 si	5’-GCAGCUCAAGAAGCACUUCAAdTdT-3’
	UCP2 si	5’-GCACCGUCAAUGCCUACAAUUdTdT-3’
	OPA1 si	5’-GUUAUCAGUCUGAGCCAGGdTdT-3’
	PRMT1 si	5’-CGUCAAACCCAACAAGUUAdTdT-3’
	control si	5’-UUCUCCGAACGUGUCACGU-3’

Supplementary table 1: **The siRNAs and primers used for experiments**. All siRNA used were purchased from Microsynth (Switzerland).
