# Supplementary material for: MICU1 controls spatial membrane potential gradients and guides Ca2+ fluxes within mitochondrial substructures
Source: Commun Biol. 2022 Jul 1;5:649. doi: 10.1038/s42003-022-03606-3 (PMC9249747; doi:10.1038/s42003-022-03606-3)
Supplement: Supplementary file 3 — Description of Additional Supplementary Files [file 42003_2022_3606_MOESM3_ESM.pdf]

Description of Additional Supplementary Files

File name: Supplementary Data

Description: Original data for main figures.
