# Supplementary material for: MICU1 controls spatial membrane potential gradients and guides Ca2+ fluxes within mitochondrial substructures
Source: Commun Biol. 2022 Jul 1;5:649. doi: 10.1038/s42003-022-03606-3 (PMC9249747; doi:10.1038/s42003-022-03606-3)
Supplement: Supplementary file 5 — Reporting Summary [file 42003_2022_3606_MOESM5_ESM.pdf]

nature portfolio

Corresponding author(s): Wolfgang Graier

Last updated by author(s): Apr 3, 2022

Reporting Summary

Nature Portfolio wishes to improve the reproducibility of the work that we publish. This form provides structure for consistency and transparency in reporting. For further information on Nature Portfolio policies, see our Editorial Policies and the Editorial Policy Checklist.

Statistics

For all statistical analyses, confirm that the following items are present in the figure legend, table legend, main text, or Methods section.

n/a	Confirmed	
☐	☒	The exact sample size (*n*) for each experimental group/condition, given as a discrete number and unit of measurement
☐	☒	A statement on whether measurements were taken from distinct samples or whether the same sample was measured repeatedly
☐	☒	The statistical test(s) used AND whether they are one- or two-sided<br>*Only common tests should be described solely by name; describe more complex techniques in the Methods section.*
☐	☒	A description of all covariates tested
☐	☒	A description of any assumptions or corrections, such as tests of normality and adjustment for multiple comparisons
☐	☒	A full description of the statistical parameters including central tendency (e.g. means) or other basic estimates (e.g. regression coefficient) AND variation (e.g. standard deviation) or associated estimates of uncertainty (e.g. confidence intervals)
☐	☒	For null hypothesis testing, the test statistic (e.g. *F*, *t*, *r*) with confidence intervals, effect sizes, degrees of freedom and *P* value noted<br>*Give P values as exact values whenever suitable.*
☒	☐	For Bayesian analysis, information on the choice of priors and Markov chain Monte Carlo settings
☒	☐	For hierarchical and complex designs, identification of the appropriate level for tests and full reporting of outcomes
☐	☒	Estimates of effect sizes (e.g. Cohen's *d*, Pearson's *r*), indicating how they were calculated

Our web collection on statistics for biologists contains articles on many of the points above.

Software and code

Policy information about availability of computer code

Data collection	NIS-elements(4.50), Visiview(2.0.8), live acquisition software (2.0.0.12)
Data analysis	ImageJ (1.53g), Microsoft Excel Professional Plus 2013, NIS-elements(5.20), Graphpad Prism (5.01)

For manuscripts utilizing custom algorithms or software that are central to the research but not yet described in published literature, software must be made available to editors and reviewers. We strongly encourage code deposition in a community repository (e.g. GitHub). See the Nature Portfolio guidelines for submitting code & software for further information.

Data

Policy information about availability of data

All manuscripts must include a data availability statement. This statement should provide the following information, where applicable:

- Accession codes, unique identifiers, or web links for publicly available datasets
- A description of any restrictions on data availability
- For clinical datasets or third party data, please ensure that the statement adheres to our policy

The data that support the findings of this study are available from the authors on reasonable request, see author contributions for specific data sets.

Field-specific reporting

Please select the one below that is the best fit for your research. If you are not sure, read the appropriate sections before making your selection.

☒ Life sciences ☐ Behavioural & social sciences ☐ Ecological, evolutionary & environmental sciences

For a reference copy of the document with all sections, see nature.com/documents/nr-reporting-summary-flat.pdf

Life sciences study design

All studies must disclose on these points even when the disclosure is negative.

Sample size	No statistical methods was used to predetermine the sample size.
Data exclusions	No data exclusions were done.
Replication	All attempts at replication were successful.
Randomization	Randomization was not relevant to our study.
Blinding	Due to the workflow of sample acquisition and analysis blinding was not relevant to our study.

Reporting for specific materials, systems and methods

We require information from authors about some types of materials, experimental systems and methods used in many studies. Here, indicate whether each material, system or method listed is relevant to your study. If you are not sure if a list item applies to your research, read the appropriate section before selecting a response.

Materials & experimental systems

n/a	Involved in the study	
☐	☒	Antibodies
☐	☒	Eukaryotic cell lines
☒	☐	Palaeontology and archaeology
☒	☐	Animals and other organisms
☒	☐	Human research participants
☒	☐	Clinical data
☒	☐	Dual use research of concern

Methods

n/a	Involved in the study	
☒	☐	ChIP-seq
☒	☐	Flow cytometry
☒	☐	MRI-based neuroimaging

Antibodies

Antibodies used

Histone H3 (1B1B2) Mouse mAb (#14269) Cell Signalling; CBARA1/MICU1 (D4P8Q) Rabbit mAb (#12524) Cell Signalling

Validation

Histone H3 (1B1B2)
Specificity / Sensitivity
Mouse mAb recognizes endogenous levels of total histone H3 protein.
Species Reactivity:

Human, Mouse, Rat, Monkey
Source / Purification

Monoclonal antibody is produced by immunizing animals with a peptide specific to the carboxy terminus of human histone H3 protein.
Background
Modulation of chromatin structure plays an important role in the regulation of transcription in eukaryotes. The nucleosome, made up of DNA wound around eight core histone proteins (two each of H2A, H2B, H3, and H4), is the primary building block of chromatin (1). The amino-terminal tails of core histones undergo various posttranslational modifications, including acetylation, phosphorylation, methylation, and ubiquitination (2-5). These modifications occur in response to various stimuli and have a direct effect on the accessibility of chromatin to transcription factors and, therefore, gene expression (6). In most species, histone H2B is primarily acetylated at Lys5, 12, 15, and 20 (4,7). Histone H3 is primarily acetylated at Lys9, 14, 18, 23, 27, and 56. Acetylation of H3 at Lys9 appears to have a dominant role in histone deposition and chromatin assembly in some organisms (2,3). Phosphorylation at Ser10, Ser28, and Thr11 of histone H3 is tightly correlated with chromosome condensation during both mitosis and meiosis (8-10). Phosphorylation at Thr3 of histone H3 is highly conserved among many species and is catalyzed by the kinase haspin. Immunostaining with phospho-specific antibodies in mammalian cells reveals mitotic phosphorylation at Thr3 of H3 in prophase and its dephosphorylation during anaphase (11).

Workman, J.L. and Kingston, R.E. (1998) Annu Rev Biochem 67, 545-79.
Hansen, J.C. et al. (1998) Biochemistry 37, 17637-41.
Strahl, B.D. and Allis, C.D. (2000) Nature 403, 41-5.
Cheung, P. et al. (2000) Cell 103, 263-71.
Bernstein, B.E. and Schreiber, S.L. (2002) Chem Biol 9, 1167-73.
Jaskelioff, M. and Peterson, C.L. (2003) Nat Cell Biol 5, 395-9.
Thorne, A.W. et al. (1990) Eur J Biochem 193, 701-13.
Hendzel, M.J. et al. (1997) Chromosoma 106, 348-60.
Goto, H. et al. (1999) J Biol Chem 274, 25543-9.
Preuss, U. et al. (2003) Nucleic Acids Res 31, 878-85.
Dai, J. et al. (2005) Genes Dev 19, 472-88.

CBARA1/MICU1 (D4P8Q)
Specificity / Sensitivity
Rabbit mAb recognizes endogenous levels of total CBARA1/MICU1 protein.
Species Reactivity:

Human, Mouse, Rat, Monkey
Source / Purification

Monoclonal antibody is produced by immunizing animals with a synthetic peptide corresponding to residues surrounding Lys110 of human CBARA1/MICU1 protein.
Background
CBARA1/MICU1 is a mitochondrial protein associated to the mitochondrial inner membrane that is comprised of two EF hand helix-loop-helix motifs. CBARA1/MICU1 is involved in mitochondrial calcium entry, metabolic coupling between cytosolic calcium transients, and activation of matrix dehydrogenases (1). Mitochondrial CBARA1/MICU1 is required to preserve normal mitochondrial calcium concentration below the equilibrium level by interacting with the uniporter pore-forming subunit MCU (2). CBARA1/MICU1 is important in pancreatic β-cell mitochondrial calcium uptake and sustained insulin secretion (3).

Perocchi, F. et al. (2010) Nature 467, 291-6.
Mallilankaraman, K. et al. (2012) Cell 151, 630-44.
Alam, M.R. et al. (2012) J Biol Chem 287, 34445-54.

Eukaryotic cell lines

Policy information about cell lines

Cell line source(s)	HeLa (ATCC-CCL-2.2TM)
Authentication	None of these cell lines were authentificated by us.
Mycoplasma contamination	Cell lines were regularly tested for mycoplasma contamination (negative).
Commonly misidentified lines (See ICLAC register)	No commonly misidentified lines were used.
